# Health literacy and quality of life of riverine populations in primary health care*

**DOI:** 10.1590/1518-8345.7402.4440

**Published:** 2025-03-14

**Authors:** Ana Kedma Correa Pinheiro, Rejane de Fátima Parada Viegas, Ingrid Bentes Lima, Ivaneide Leal Ataide Rodrigues, Sheila Nascimento Pereira de Farias, Laura Maria Vidal Nogueira

**Affiliations:** 1 Universidade Federal do Rio de Janeiro, Escola de Enfermagem Anna Nery, Rio de Janeiro, RJ, Brazil; 2 Scholarship holder at the Fundação Amazônia Paraense de Amparo à Pesquisa (FAPESPA), Brazil; 3 Scholarship holder at the Coordenação de Aperfeiçoamento de Pessoal de Nível Superior (CAPES), Brazil; 4 Universidade do Estado do Pará, Escola de Enfermagem Magalhães Barata, Belém, PA, Brazil; 5 Universidade do Estado do Pará, Departamento de Enfermagem Comunitária, Belém, PA, Brazil

**Keywords:** Health Literacy, Quality of Life, Socioeconomic Factors, Rural Health, Rural Nursing, Primary Health Care

## Abstract

to analyze functional health literacy and health-related quality of life in riverine populations using primary care services, according to sociodemographic variables.

an analytical, cross-sectional study with 312 users of the riverine family health strategy. Data were collected using a health literacy test, the 12-item Health Survey, and a socioeconomic questionnaire adapted by the researchers. Spearman correlation, Mann-Whitney and Kruskal-Wallis tests, as well as multiple logistic regression were performed, considering p≤0.05.

65.7% presented inadequate functional health literacy, with higher risk for men (p<0.001), aged 40-49 (p=0.010) and 50-59 years (p=0.031), incomplete (p<0.001) and complete (p=0.024) elementary education, and residing far from health services (p<0.001). Quality of life showed no association with health literacy. However, lower quality of life was related to female gender (p=0.049), incomplete elementary education (p=0.016), use of mobile phones with internet and radio (p=0.013), advanced age (p<0.001), increased number of children (p=0.002), and lower age at the start of work activities (p<0.001).

functional health literacy of riverine populations is inadequate and not associated with quality of life. However, both are influenced by the sociodemographic profile.

## Highlights

**(1)** Health literacy of riverine populations is inadequate. **(2)** Health literacy was not associated with quality of life. **(3)** Health literacy was associated with sociodemographic aspects of riverine populations. **(4)** Quality of life was related to the sociodemographic profile of riverine populations.

## Introduction

Functional Health Literacy (FHL) refers to a person’s cognitive ability to read, interpret, and decode information available and provided in health services, crucial for therapeutic decision-making and maintenance of self-care and well-being^(1)^.

This study adopted the concepts of the Health Literacy (HL) theoretical model^(2)^, which considers knowledge, motivation, and competencies for maintaining or improving Quality of Life (QoL). This model encompasses three groups of factors, from distal to proximal: socio-environmental determinants, involving demographics, culture, and social systems; situational factors involving family support and media; and personal factors encompassing socioeconomic variables.

QoL reaches a broad concept with dimensions in physical, mental, and social spheres. It is directly linked to functional capacity, socioeconomic well-being, and the person’s degree of satisfaction. The physical and mental components incorporate important variables to assess Health-Related Quality of Life (HRQoL)^(3)^.

This topic arouses global interest, ratified by scientific evidence from a search conducted in seven databases, including the Medical Literature Analysis and Retrieval System Online. Various studies addressing HL and QoL were identified; however, only five involved rural populations, and none involved riverine populations.

Among studies associating HL and QoL, positive repercussions were evident in relation to self-perception of health status^(4-9)^, self-management^(10-14)^, health promotion-focused interventions^(15-16)^, treatment adherence^(17)^, physical health^(18-19)^, and mental health^(20-22)^. Negative repercussions were related to lack of access to health information, worsening of clinical conditions, and an increased demand for medical support^(23-24)^.

A study conducted in Germany^(4)^ with people served in an integrated health system, similar to the Primary Health Care (PHC) context, confirmed an association between adequate HL and high HRQoL in rural areas. Despite the geographical characteristics of rural areas, these populations are not considered riverine.

Another initiative concerns a study conducted in China^(20)^ with people in a pre-diabetic state in the PHC context, which identified an association between adequate HL and high HRQoL Although conducted with rural area residents, it did not involve riverine communities.

In Brazil, in the state of Pará, research addressing FHL revealed an association between inadequate literacy and low education and income levels, confirming repercussions on the self-care deficit and health promotion of PHC users^(1)^. It is noteworthy that Pará is part of the Brazilian Amazon, home to a significant riverine population, whose life context is peculiar and may impact morbidity and mortality indicators, requiring careful observation of the sociodemographic profile and way of life^(25)^.

Riverine populations depend almost exclusively on the Unified Health System (SUS) and seek the service closest to their residence. However, they are constrained to environments with difficult locomotion and precarious means of transport. The work of PHC teams in these communities needs to ensure resoluteness; however, they face geographical challenges and a reduced number of professionals with high turnover due to fragile labor ties^(26)^.

It should be emphasized that the actions of PHC teams and health managers need to be guided by the National Policy for the Comprehensive Health of Populations from Rural, Forest, and Water Areas^(27)^ and the National Policy for Primary Care (PNAB)^(28)^ to meet the specific needs of local residents. In the work routine, it is challenging and essential for health professionals to use vocabulary and educational materials comprehensible to these populations, which refers to the field of HL^(29)^.

Thus, the approach of this study is of great interest to the advancement of science, as it presents scientific evidence on a vulnerable population with a unique way of life and the potential for replication in other geographic areas that share characteristics similar to riverine communities, especially in Latin America.

To better understand the relationship between FHL and HRQoL in an Amazonian riverine community, this study aimed to analyze functional health literacy and health-related quality of life in a riverine population using primary care services, according to sociodemographic variables.

## Method

### Study design

This analytical and cross-sectional study was based on the Strengthening the Reporting of Observational Studies in Epidemiology (STROBE)^(30)^ as a guideline for manuscript writing.

### Study locus and population

It was conducted in a riverine area of the municipality of Abaetetuba, Pará state, Brazil, located in the Lower Tocantins region, with a population of 158,188 according to the 2022 census^(31)^. The riverine region encompasses 72 localities, with approximately 42,000 inhabitants^(32)^.

The study was carried out in the territory assigned to the Riverine Family Health team (RFHt) that serves users from four localities, referred to in this study as rivers: X, Y, Z, W. The RFHt is located on river X and covers eight micro-areas, two of which lack Community Health Agents (CHA) coverage, belonging to river Z. The choice of locations was based on the population served by the only RFHt in the region.

According to the Unified Health System Territorialization System (*e-SUS-território*) registry, river X has 461 inhabitants aged ≥18 years, river Y has 133, river Z has 118, and river W has 668, totaling 1,380 users served by the RFHt. Additionally, river Z has unquantified residents who are not registered in *e-SUS-território* due to residing in geographical areas not assisted by CHAs.

### Selection criteria

The study included users aged ≥18 years, of both sexes, who could read and write, residing in the riverine region of Abaetetuba municipality, within the RFHt’s assigned territory. Exclusion criteria were individuals with visual and/or auditory limitations that prevented reading the instruments or hearing the interviewer during data collection, as well as those with cognitive or behavioral insufficiency that hindered study participation, previously identified in *e-SUS-território* records and/or self-reported by participants or family members.

### Definition of the sample

The sample consisted of 312 users, defined using Cochran’s sampling technique^(33)^, considering the population size and a 5% sampling error.

Representatives from all four rivers were included proportionally, considering user registration in *e-SUS-território*. Different education strata were also considered, according to data from the Brazilian Institute of Geography and Statistics (IBGE), recognizing the following proportions: 38.6% of the population with no education or incomplete elementary education; 12.5% with complete elementary and incomplete high school education; and 48.8% with complete high school education or higher^(34)^.

Non-probabilistic quota sampling^(35)^ was used, as participant selection was based on proportional representation of the four localities, observing the number of residents in the territory corresponding to the three education strata. This option allowed obtaining representative data from each river.

The inclusion of all four localities was to encompass population diversity. Stratification by education level was used to avoid sampling bias that could affect literacy levels, as education has shown a relationship with HL^(29,36)^.

### Instruments used and study variables

The Health Literacy Test (HLT)^(36-37)^, validated in Brazil in 2019 and cross-culturally adapted to Brazilian Portuguese^(36-37)^, was used. It consists of two parts: assessment of numeracy domain with 17 items and assessment of reading and comprehension with 50 items. This test was chosen for its ability to evaluate both domains. Internal consistency was 0.953, and its application followed the authors’ recommendations^(36-37)^.

HLT scores followed the original instrument’s recommendations^(37)^. For calculation, one point was assigned for each correct answer and zero for incorrect answers, self-declared “don’t know”, or non-completion. For the numerical part, the assigned score, up to 17 in the raw score, was multiplied by 2.941 to convert to a score of 0 to 50^(37)^.

The reading part had no weighting. Final scores were calculated by summing the domains^,^ classifying adequate literacy as 75-100 points, limited literacy as 60-74 points, and inadequate literacy as 0-59 points^(36)^.

To measure Health-Related Quality of Life (HRQoL), the 12-item Health Survey (SF-12), version 1.0, created in 1994 and validated in Brazil in 2004, was used^(39)^. It consists of 12 items divided into two components: the Physical Component Summary (PCS) and the Mental Component Summary (MCS). Each component has four domains: PCS involves general health, pain, functional capacity, and physical aspects; MCS encompasses emotional aspects, mental health, vitality, and social aspects^(39)^.

SF-12 was chosen for being a simple, short, quick-to-apply instrument widely used to accurately and efficiently measure physical and mental health in general population research. It has a high degree of reliability, with a Cronbach’s alpha coefficient of 0.836^(40-41)^.

SF-12 score calculation followed the original instrument’s recommendations^(38)^, using a graduated Likert-type scale with scores varying according to question composition, with two to six response options, ranging from 0 to 100, with higher scores associated with better QoL levels. Each component was analyzed separately, without establishing a total score^(38-39)^, but they were grouped into PCS and MCS. To define the component scores, the scoring algorithm was considered, using the 1998 U.S. population mean, which has been adopted in Brazilian and international studies^(38-41)^.

To outline the sociodemographic profile, a questionnaire developed by the research nucleus and national confederation of agricultural workers was used, structured for the “Itinerant Listening: Access of Rural and Forest Populations to SUS” project^(42)^. The instrument has two parts: the first concerns profile and lifestyle; rural production and work; and the health system. The second part corresponds to participants’ sex and age identity.

Seven questions related to this study were extracted from this instrument, allowing data on the sociodemographic profile of riverine populations to be obtained. These questions are included in the first part of the questionnaire, with six referring to profile/lifestyle, including sex, age, locality, education, number of children, and means of communication. The other question concerns rural production/work, which allowed investigation of the age at which work activities began. These variables were adapted in light of research conducted with Amazonian riverine populations^(3,43)^.

A pilot study was conducted with riverine PHC users residing in other localities of the municipality, which allowed adapting HLT terms to local culture considering semantic meaning. It was also important for directing interviewer training and planning fieldwork.

The adjustments made to the HLT refer to the insertion of some explanatory terms for technical words to facilitate participants’ understanding. In the numeracy test, words were inserted to explain the following terms: capsule = pill; antipyretic = medicine to treat fever; and Tylenol = paracetamol. In the reading comprehension test, the following explanations were added: person who has asthma = asthmatic; heartburn medicine = antacid; bone disease = osteoporosis; low blood sugar = hypoglycemia; and mental illness = schizophrenia.

### Data collection

Data collection occurred between February and May 2021, at the RFHt building while waiting for care and during home visits scheduled by CHAs, at the participants’ discretion. The average duration of interviews was twenty-three minutes.

The HLT was self-completed in the comprehension and reading part, where the interviewee filled in the blanks by selecting the most appropriate possibility for the text, evaluated by the following sections: Section A contained instructions for the radiography procedure, section B included the initial text of Brazilian legislation concerning SUS, and section C consisted of a consent form for procedures. The numeracy part was assisted by the interviewer, who provided cards with numerical information and subsequently asked the questions provided in the instrument. The application time for each part of the HLT was recorded^(36)^. The SF-12 was administered with the interviewer’s assistance.

The interviewers were the main researcher and five undergraduate nursing students from a state public university, enrolled in the Nursing and Traditional Amazonian Populations course. They were previously trained and supervised by the main researcher.

Obstacles encountered during data collection involved logistical limitations, such as: navigating small silted rivers with tidal waves, passing through narrow channels, difficulty allocating safe transportation for the team, and conflicting schedules between data collection and riverine residents’ work hours.

To overcome these, the research team received support from community leaders who housed the researchers, enabling them to stay in the territory and make better use of data collection time. This collaboration also resulted in the hiring of a medium-sized vessel capable of handling tidal waves.

### Data treatment and analysis

Data were entered with double entry into the Statistical Package for the Social Sciences, version 21. Spearman’s correlation was chosen to compare scores and ages. The Shapiro-Wilk test was used to assess normality. For bivariate analysis of these scores with sociodemographic variables, Mann-Whitney and Kruskal-Wallis tests were applied, allowing comparison of scores with categorical variables.

A logistic regression model was used to measure the association between variables of interest (sex, age, location, education, number of children, means of communication, and start of work life) and the outcome of inadequate FHL. Variables with significant association in the bivariate logistic regression analysis remained in the adjusted model. All analyses were performed using the R statistical package, version 4.1.1, considering a significance level of 0.05.

### Ethical aspects

The study followed Resolutions 466/12 and 580/18 of the National Health Council/Ministry of Health and was approved by the Research Ethics Committee of the Undergraduate Nursing Course at the State University of Pará, under opinion 4,517,829 of February 1, 2021.

## Results

Of the 312 riverine residents in the sample, 67.0% (n=209) were female, and most had two to three children, with an average of 2.18±1.999, ranging from zero to 13 children. The mean age was 35.03±13.397 years, ranging from 18 to 78 years, with the highest frequency (71.5%) between 18 and 39 years. Of the total sample, 61.5% used mobile phones (Table 1).

The mean age for starting work life was 12.51±4.518 years, ranging from 3 to 31 years, with half the sample starting activities between 3 and 13 years old (n=156; 50.0%). The mean total score of the HLT was 52.235±21.638, ranging from 5.941-99.997. Of the total, 205 (65.7%) presented inadequate FHL, 54 (17.3%) limited, and 53 (17.0%) adequate. The mean total FHL score (p<0.001) showed a reduction proportional to the impairment of literacy. Higher mean FHL scores were identified in females (p<0.001), residents of river X (p<0.001), and mobile phone users with internet access (p<0.001). Lower means were identified among residents of river W (p<0.001) without access to communication devices (p<0.001). FHL decreased with an increase in the number of children (p<0.001) and age (p<0.001), and increased with higher education levels (p<0.001) and age of starting work activities (p<0.001) (Table 1).

**Table 1 -:** Analysis of Functional Health Literacy according to sociodemographic variables of riverine residents (n* = 312). Abaetetuba, PA, Brazil, 2021

			**HLT** ^†^ **score**		
**Variable**	**Category**	n* (%) ^‡^	**Mean**	**Standard Error**	**p-value** ^§^
**Gender**					
	Female	209 (67.0%)	55.530	1.531	<0.001
	Male	103 (33.0%)	45.551	1.873	
**Number of children**					
	0	61 (19.6%)	59.157	2.723	<0.001
	1	66 (21.2%)	56.756	2.353	
	2 - 3	123 (39.4%)	51.940	1.997	
	4 - 13	58 (18.6%)	40.563	2.540	
	NR ^||^	4 (1.3%)	50.410	10.754	
**Age (years old)**					
	18 - 29	122 (39.1%)	59.462	1.816	<0.001
	30 - 39	101 (32.4%)	53.331	2.178	
	40 - 49	37 (11.9%)	44.906	3.079	
	50 - 59	24 (7.7%)	38.150	4.291	
	60-78	24 (7.7%)	35.775	3.078	
	NR ^||^	4 (1.3%)	55.190	10.176	
**Locality**					
	River X	111 (35.6%)	59.276	2.118	<0.001
	River Y	33 (10.6%)	49.232	3.282	
	River Z	29 (9.3%)	53.783	3.725	
	River W	139 (44.6%)	47.003	1.747	
**Schooling**					
	IEE ^¶^	132 (42.3%)	37.432	1.383	<0.001
	CEE**	32 (10.3%)	53.138	3.135	
	IHE ^††^	30 (9.6%)	56.120	3.164	
	CHE ^‡‡^	85 (27.2%)	64.998	1.854	
	IHrE ^§§^	15 (4.8%)	78.978	4.232	
	CHrE ^||||^	18 (5.8%)	70.158	4.443	
**Communication**					
	Mobile phone	192 (61.5%)	46.839	1.434	<0.001
	Mobile phone and internet	110 (35.3%)	62.982	1.995	
	Mobile, internet, and radio	3 (1.0%)	44.743	9.127	
	No	3 (1.0%)	39.508	7.172	
	NR ^||^	4 (1.3%)	30.881	8.662	
**Start of work life**					
	3 - 13 years	156 (50.0%)	45.240	1.544	<0.001
	14 - 17 years	60 (19.2%)	52.62	2.629	
	9 - 31 years old	39 (12.5%)	67.310	3.038	
	Never worked	55 (17.6%)	61.030	3.081	
	NR ^||^	2 (0.6%)	50.440	20.794	

*n = Number of participants; ^†^HLT = Health Literacy Test; ^‡^% = Percentage frequency; ^§^p-value = Mann-Whitney test for variables with two categories and Kruskal-Wallis for others, significant at p≤0.05; ^||^NR = Not Reported; ^¶^IEE = Incomplete Elementary Education; **CEE = Complete Elementary Education; ^††^IHE = Incomplete High School Education; ^‡‡^CHE = Complete High School Education; ^§§^IHrE = Incomplete Higher Education; ^||||^CHrE = Complete Higher Education

Regarding HRQoL, the PCS and MCS presented means of 43.461±10.176 and 47.752±9.633, with medians of 44.730 and 50.191, ranging from 14.063-62.075 and 19.317-66.982, respectively. PCS (p=0.090) and MCS (p=0.776) showed no association with FHL. However, females (p=0.049) presented a statistically significant difference with lower HRQoL in MCS (Table 2).

Regarding PCS, a lower HRQoL was identified in participants with incomplete elementary education (p=0.016) and those who used mobile phones with internet and radio (p=0.013). However, those who did not use communication devices showed better QoL in PCS (p=0.013). The PCS of HRQoL decreased with an increase in the number of children (p=0.002), advancing age up to 59 years (p<0.001), and lower age at the start of work activities (p<0.001) (Table 2).

**Table 2 -:** Analysis of health-related quality of life according to functional health literacy and sociodemographic characteristics of riverine residents (n* = 312). Abaetetuba, PA, Brazil, 2021

		**PCS** ^†^			**MCS** ^‡^		
**Variable**	**Category**	**Mean**	**Standard Error**	**p-value** ^§^	**Mean**	**Standard Error**	**p-value** ^§^
**FHL** ^||^							
	Inadequate	42.495	0.749	0.090	47.744	0.667	0.776
	Limited	45.724	1.246		48.239	1.424	
	Adequate	44.892	1.161		47.285	1.267	
**Gender**							
	Female	43.346	0.737	0.813	47.042	0.655	0.049
	Male	43.695	0.903		49.192	0.970	
**Number of children**							
	0	46.212	1.044	0.002	46.674	1.216	0.084
	1	46.235	1.161		49.644	1.237	
	2 - 3	42.218	0.949		48.063	0.859	
	4 - 13	40.145	1.446		46.603	1.218	
	Not reported	42.046	4.062		40.040	4.766	
**Age (years old)**							
	18 - 29	46.197	0.835	<0.001	48.121	0.887	0.180
	30 - 39	44.823	0.919		47.779	0.917	
	40 - 49	39.204	1.767		46.036	1.562	
	50 - 59	34.553	2.197		50.625	1.844	
	60-78	39.388	1.935		44.526	2.275	
	Not reported	42.899	5.886		53.780	2.088	
**Schooling**							
	IEE ^¶^	41.241	0.901	0.016	47.605	0.845	0.364
	CEE**	43.790	2.066		45.994	1.620	
	IHE ^††^	46.978	1.657		48.250	1.488	
	CHE ^‡‡^	44.883	0.998		48.710	1.099	
	IHrE ^§§^	46.860	2.287		44.267	2.590	
	CHrE ^||||^	43.750	2.526		49.504	2.240	
**Communication**							
	Mobile phone	42.006	0.763	0.013	47.991	0.697	0.393
	Mobile phone and internet	45.876	0.853		47.874	0.904	
	Mobile, internet, and radio	37.264	5.894		38.000	7.060	
	No	54.009	3.015		43.177	5.167	
	Not reported	43.647	5.366		43.650	5.578	
**Start of work life**							
	3 - 13 years	40.964	0.877	<0.001	47.468	0.797	0.816
	14 - 17 years	44.943	1.178		47.189	1.317	
	9 - 31 years old	45.213	1.473		49.666	1.389	
	Never worked	47.850	1.032		47.881	1.157	
	Not reported	38.956	1.476		45.914	13.167	

*n = Number of participants; ^†^PCS = Physical Component Summary; ^‡^MCS = Mental Component Summary; ^§^p-value = Mann-Whitney test for variables with two categories and Kruskal-Wallis for others, significant at p≤0.05; ^||^FHL = Functional Health Literacy; ^¶^IEE = Incomplete Elementary Education; **CEE = Complete Elementary Education; ^††^IHE = Incomplete High School Education; ^‡‡^CHE = Complete High School Education; ^§§^IHrE = Incomplete Higher Education; ^||||^CHrE = Complete Higher Education

Table 3 indicates that the better the PCS, the higher the reading ability (p≤0.01), and the higher the scores in sections A (p≤0.05) and B (p≤0.05) of the reading test. However, the worse the PCS, the higher the age (p≤0.01), and the more time used in completing section A (p≤0.05) and the total reading sections (p≤0.05). There was no significant correlation between the MCS and the HLT measurement items.

**Table 3 -:** Spearman’s correlation between the physical and mental components of health-related quality of life with age and health literacy test of riverine residents (n* = 312). Abaetetuba, PA, Brazil, 2021

**Variables**	**PCS** ^†^	**MCS** ^‡^
Age	-0.334 ^§^	-0.059
Time for reading section A	-0.172 ^||^	-0.019
Time for reading section B	0.053	0.021
Time for reading section C	0.09	0.011
Total reading time	-0.152 ^||^	-0.025
Score for reading section A	0.159 ^||^	0.055
Score for reading section B	0.150 ^||^	0.065
Score for reading section C	0.142	-0.001
Total reading score	0.189 ^§^	0.073
Numeracy score	0.06	0.082
Time for numeracy test	-0.115	0.017
Total score of the health literacy test	0.140	0.084

*n = Number of participants; ^†^PCS = Physical Component Summary; ^‡^MCS = Mental Component Summary; ^§^≤0.01; ^||^>0.01 - ≤0.05

The bivariate and adjusted models are presented in Table 4. The model adjustment indicates that the use of mobile phones with internet access may be a protective factor against inadequate FHL. Furthermore, being male, aged between 40 to 49 and 50 to 59 years, living farther from health services (River W), and having completed only elementary education (incomplete or complete) increased the risk of inadequate FHL.

**Table 4 -:** Association of inadequate Health Literacy with sociodemographic characteristics (n* = 312). Abaetetuba, PA, Brazil, 2021

	**Bivariate model**		**Adjusted model**	
**Characteristics**	**OR** ^†^ **(** CI **95%)** ^‡^	**p-value** ^§^	**OR** ^†^ **(** CI **95%)** ^‡^	**p-value** ^§^
Sex (reference = Female)				
Male	2.96 (1.72; 5.29)	<0.001	4.42 (2.11; 9.73)	<0.001
Number of children (reference = 0 children)				
1 child	1.32 (0.66; 2.66)	0.438		
2 or 3 children	2.5 (1.33; 4.74)	0.005		
4 children or more	4.79 (2.16; 11.3)	<0.001		
Age (reference = 18 to 29 years old)				
30-39 years old	1.75 (1.02; 3.01)	0.043	1.51 (0.72; 3.20)	0.278
40-49 years old	7.98 (2.95; 28.0)	<0.001	6.01 (1.68; 26.4)	0.010
50-59 years old	6.77 (2.19; 29.7)	0.003	5.92 (1.29; 34.6)	0.031
60+ years old	10.6 (2.96; 68.3)	0.002	3.66 (0.67; 31.9)	0.174
Not reported	0.97 (0.11; 8.28)	0.974	0.94 (0.05; 14.3)	0.963
Locality (reference = River X)				
River Y	2.18 (0.97; 5.19)	0.066	1.81 (0.61; 5.62)	0.290
River Z	1.55 (0.68; 3.67)	0.305	1.6 (0.45; 5.74)	0.468
River W	3.17 (1.85; 5.50)	<0.001	3.7 (1.78; 7.94)	<0.001
Education level (reference = Complete Higher Education)				
Incomplete Higher Education	0.31 (0.04; 1.64)	0.195	0.43 (0.05; 2.91)	0.410
Complete Elementary School	5.11 (1.53; 18.9)	0.010	5.61 (1.31; 26.8)	0.024
Incomplete Elementary School	31 (9.67; 112)	<0.001	16.5 (4.35; 70.3)	<0.001
Complete High School	1.21 (0.42; 3.76)	0.731	1.03 (0.29; 3.99)	0.959
Incomplete High School	3 (0.91; 10.8)	0.078	2.91 (0.69; 13.4)	0.156
Means of communication (reference = Mobile phone)				
Mobile phone and internet	0.25 (0.15; 0.40)	<0.001	0.47 (0.23; 0.94)	0.033
Mobile phone, internet, and radio	0.61 (0.06; 13.4)	0.692	2.47 (0.09; 149)	0.645
No	4791233 (0.00; Inf ^||^ )	0.986	3900155 (0.00; Inf ^||^ )	0.985
Start of work life (reference = 3 to 13 years old)				
14 - 17 years	0.6 (0.31; 1.20)	0.141		
9 - 31 years old	0.13 (0.06; 0.27)	<0.001		
Never worked	0.22 (0.11; 0.41)	<0.001		

*n = Number of participants; ^†^OR = Odds Ratio*;* ^‡^CI = Confidence Interval (95%); ^§^p-value = Mann-Whitney test for variables with two categories and Kruskal-Wallis for others, significant at p≤0.05; ^||^Inf = Infinity

## Discussion

Functional Health Literacy (FHL) was found to be inadequate in most participants. In this regard, a study conducted in Brazil^(36)^ that validated the Health Literacy Test (HLT) identified that approximately half of the 302 participants had inadequate or limited FHL, justified by sociodemographic factors, especially increasing age and fewer years of education.

In contrast, research conducted in rural areas of Germany concluded that 61.9% had sufficient HL. Although the sample comprised population groups with unfavorable social and educational profiles, the HL level was explained by investment in prevention, health promotion, and management empowerment, producing a positive effect on people^(4)^.

In light of these discussions and the sociodemographic profile of riverine residents, the prevalence of inadequate basic numeracy and reading comprehension skills, necessary for understanding health information, is justified. The distance and logistical limitations to accessing health services may interfere with FHL performance. Moreover, social vulnerabilities present in the local context can impact the use of information provided by health services and affect individual decisions.

In this sense, the higher proportion of women was similarly reported in national and international HL literature, indicating a predominance of females^(1,15,36,44-45)^. This can be attributed to women seeking health services more often and the absence of men in family environments during data collection due to work activities.

Amazonian riverine women are expected to play an important role in the family care and support network, accumulating unpaid household activities, with a profile of low education, teenage pregnancy, and high gestational and fertility rates. These results can guide the construction of relevant therapeutic projects in the context of HL for Amazonian riverine women^(43)^.

Regarding FHL, women showed better levels of numeracy and reading comprehension skills, as well as a lower risk of inadequate FHL, which is consistent with a study conducted in Japan^(45)^, where women demonstrated better HL ability for decision-making. Although the study was conducted in another country, similarities can be seen with the riverine context of the Pará Amazon, where women assume the role of primary family caregiver, being more likely to apply health promotion measures, in addition to managing appointments and medications, and having a greater presence in health services.

On the other hand, women showed greater impairment in mental QoL, highlighting the dimension of the female universe that involves activities peculiar to the patriarchal model, where women are responsible for childcare and household duties, situations that impact emotional and psychological stability, potentially triggering depressive states^(46)^.

Regarding fertility, it was identified that the average number of children is equivalent to the population replacement value of 2.1 children per woman, surpassing the figures recorded in Brazil, corresponding to 1.4 children per woman, according to a United Nations research, which admitted higher fertility rates among people with lower education, poorer, from rural areas, and from the northern region of Brazil^(47)^.

An inverse relationship was found between FHL and the number of children, as the fewer children, the better the FHL, and those without children achieved even higher FHL. In this regard, a health survey conducted in riverine communities in Amazonas^(43)^ detected that women had their first pregnancies during adolescence and a high fertility. This should be considered for adopting reproductive education measures under the theoretical framework of HL to achieve a better QoL.

The assessment of physical QoL allowed the identification of an association with the number of children due to the demands of caregiving combined with work activities and household tasks that require physical effort. In this sense, a systematic review study reported a negative influence of the number of children on the QoL of family caregivers when related to emotional aspects and vitality^(48)^.

Regarding age peculiarities, it was found that the frequency of participants decreased with increasing age, with a higher proportion of young adult participants, approaching the average age obtained in an HL and QoL study conducted in rural and urban areas of China^(49)^. Furthermore, an inverse relationship between age and HL was evident, meaning that the more advanced the age, the worse the HL and the higher the risk of inadequate FHL, converging with research conducted in Brazil on the influence of HL in patients with coronary artery disease^(50)^.

This scenario may result from limitations inherent to the aging process, which can cause impairment in the ability to assimilate information, influenced by variables such as years of education and reading habits. As age increases, cognitive capacity is affected, decreasing the ability to understand health knowledge^(45,51)^, resulting from limitations in understanding illness conditions and the working methods of riverine residents, including: low education, exposure to ergonomic risks resulting from activities that require great physical effort, inadequate posture, prolonged working hours, monotony, repetitiveness, and imposition of intense routines.

The absence of schools in some localities and restricted access to public higher education can potentiate limited health knowledge, considered a historical remnant of precarious and less accessible education, especially for vulnerable people^(52)^.

Thus, education is directly related to HL; the higher the education level, the better the HL. Lower education presents greater risks of inadequate FHL and is related to the country’s development level, considering that the higher the development, the better the education and HL indices^(53)^.

In this study, people residing farther from PHC services showed worse FHL and a higher risk of inadequate FHL compared to those living in the locality where the service is implemented, which can be attributable to difficult access to the service and, consequently, less demand for care. Therefore, attending health services significantly impacts the FHL level of these populations^(54)^. Limited access to information has been associated with unfavorable health conditions and contexts^(55)^.

The most distant locality, although covered by CHAs, is a region known for its tidal waves and difficult travel conditions due to heavy boat traffic on the route to the RFHt. Riverine populations face significant limitations in accessing health services due to unique accessibility challenges and geographical obstacles. These include the presence of tidal waves, lack of reliable river transportation, and the dynamic nature of tidal patterns^(32)^. This reality contributes to the exclusion of these populations from health actions and services, highlighting the need for PHC interventions to improve HL and QoL levels^(26)^.

It is crucial to value the health-disease process of riverine residents within the context of their cultural diversity and way of life^(29)^. In this regard, it is essential for PHC to understand local specificities and culture to promote people’s HL, providing relevant information recognized as significant to their reality. This approach can enhance well-being, treatment adherence, and consequently, promote QoL.

In the peculiarities of riverine life, it was found that the most used means of communication is the mobile phone, with or without internet access. This aligns with the Brazilian Institute of Geography and Statistics (IBGE) research, which in 2019 confirmed the presence of mobile phones in 94.0% of households nationwide. It should be noted that there are restrictions on internet use in rural areas, mainly due to the difficulty of acquiring compatible devices because of high costs and unavailability of internet signals in residential areas^(56)^.

Furthermore, many riverine communities, lacking stable electricity, have limited access to information^(25)^. Access to more technologically advanced means of communication favored the FHL of riverine residents, being considered a protective factor against inadequate FHL. Those who used mobile phones with internet access had better FHL than those without devices or those using only the mobile phone.

Due to the rapid development of electronic communication technology and the range of information linked to it, it is necessary to improve digital HL or eHealth literacy. This will equip riverine residents to access and use relevant health information through electronic media^(15)^.

Physical QoL was more compromised among those who used radio and mobile phones with internet access, and less among those without communication devices. This suggests that the advancement of information technology in rural regions, coupled with excessive use, generates technological dependence and may lead to physical inactivity. This situation can be intensified by limited interaction environments and leisure spaces in the territory, as well as the physical distancing produced by the COVID-19 pandemic, factors that interfere with physical health.

It is a fact that the transformation brought by the internet reflects in reduced community interaction. However, it enables the incorporation of care technologies, such as telehealth and teleconsultations. These technologies bridge geographical distances, contributing to the effectiveness and resoluteness of assistance, especially in areas with difficult access to health services, such as Amazonian riverine communities. This can improve HL levels and positively impact physical QoL^(57)^.

In the context of riverine life, the early work of children and adolescents stands out, resulting from the denial and naturalization of child labor exploitation under the guise of increasing family income and providing opportunities for practical learning^(58)^. Nevertheless, governmental measures are distributed contradictorily in relation to child labor levels in Brazilian states. Inspections are insufficient, and income transfer policies show limitations in combating child labor^(59)^.

Child labor was related to lower QoL in the PCS, resulting from exposure to activities requiring great physical effort, such as subsistence agriculture and artisanal fishing. Such exposure can have consequences on physical health, as it involves musculoskeletal system disorders and work accidents, impacting QoL^(60)^.

When analyzing the age of start of work activities, a direct relationship with FHL was found. Children and adolescents who entered the workforce early exhibited worse HL compared to those who started as apprentices or from 18 years of age. This is due to intellectual impairment expressed in low knowledge, low motivation, and reduced numerical and reading skills, leading to school dropout and compromised health decision-making^(61)^.

Health Literacy (HL) is identified as a key action area to achieve the 2030 sustainable development agenda, addressed in the eighth sustainable development goal regarding the need for better working conditions and economic growth. Higher HL levels equip rural workers to demand better opportunities and working conditions^(61)^.

Regarding work and income opportunities, these are tied to water and forest resources that constitute their means of subsistence. They live off family agriculture and artisanal fishing, which, combined with culture, shape their subjectivity. Although capitalist logic may consider it insufficient and unsustainable, fishing and agriculture produce food sovereignty and supply regional commerce^(25)^.

In this investigation, the components of HRQoL were not significantly associated with different levels of HL, which was also identified in other studies that addressed HL and QoL, namely: a study that measured the impact of HL in patients undergoing renal replacement therapy, associated with cognitive aspects, medication adherence, and QoL^(62)^; a study conducted with vulnerable populations that examined HL in the control of diabetes in Korean Americans^(10)^; a study that evaluated the relationship between HL and people’s fear related to Coronavirus Disease 2019 (COVID-19)^(63)^; and a study that examined the relationship between HL, health-promoting lifestyles, and QoL among university students^(64)^.

On the other hand, international initiatives have identified this relationship, as in a study that measured the interrelationships between demographics, HL, self-perception of health status, and QoL in Korean and Vietnamese immigrants^(5)^; a study that correlated HL with QoL and health status in rural communities in Germany^(4)^; and research that analyzed HRQoL in adolescents and their parents with HL in the context of COVID-19^(65)^.

The sociodemographic portrait of riverine residents reveals the need for HL mapping to optimize health knowledge and communication, and to identify difficulties in understanding information received in PHC services. Therefore, it is important for the service to recognize HL as a social determinant of health necessary for understanding people’s sociodemographic conditions and way of life.

It is acknowledged that FHL investigation with vulnerable people is still poorly described in national and international literature, limiting result comparisons. Another limitation refers to the data collection instrument that includes self-completion texts unfamiliar to riverine reality, which may have caused filling bias.

However, this study contributes by offering subsidies for local and regional health planning and PHC educational actions, especially for nursing, aiming to implement practices compatible with users’ FHL levels, enabling improved QoL.

The research may contribute to promoting service interventions and encourage the inclusion of HL approaches in undergraduate and postgraduate health curricula. It may also favor potential advancement in the National Policy for the Comprehensive Health of Populations from Rural, Forest, and Water Areas by incorporating an intersectoral perspective to promote HL and QoL, articulating traditional knowledge, public health, and education policies. Thus, equitable and resolutive provision of health education actions is envisioned through strategies appropriate to people’s way of life.

## Conclusion

This pioneering study in Brazil, which evaluated FHL and HRQoL of riverine PHC users, showed no association between FHL and the PCS and MCS of HRQoL. However, it identified congruence of FHL and HRQoL with sociodemographic aspects.

Riverine PHC users presented inadequate FHL, pointing to the need for effective implementation of specific public policy and strategic planning to offer significant health information appropriate to these populations’ sociodemographic profile, to impact health self-management.

The sociodemographic portrait of riverine residents reveals that men, elderly, those living farther from services, without internet access, with lower education, and who suffered child labor exploitation presented worse FHL, requiring simple, objective, and meaningful health communication from PHC.

For interventions to improve riverine residents’ QoL, it is necessary to consider that their profile shows physical QoL with greater impairment in the elderly, those with low education, access to communication means, higher fertility rates, child labor exploitation, and women who had worse mental QoL.

Overall, this study showed that the main association variables relate to sociodemographic aspects that interfered with reading and numeracy skills, and the PCS and MCS of riverine PHC users.
