# Supplementary material for: Health literacy and quality of life of riverine populations in primary health care
Source: Rev Lat Am Enfermagem. 2025 Mar 14;33:e4440. doi: 10.1590/1518-8345.7402.4440 (PMC11912821; doi:10.1590/1518-8345.7402.4440)
Supplement: Translation

# Translation: Letramento funcional em saúde e qualidade de vida de ribeirinhos na atenção primária à saúde*

**DOI:** 10.1590/1518-8345.7402.4441

**Authors:** Ana Kedma Correa Pinheiro, Rejane de Fátima Parada Viegas, Ingrid Bentes Lima, Ivaneide Leal Ataide Rodrigues, Sheila Nascimento Pereira de Farias, Laura Maria Vidal Nogueira

**Affiliations:** 1 Universidade Federal do Rio de Janeiro, Escola de Enfermagem Anna Nery, Rio de Janeiro, RJ, Brasil; 2 Bolsista da Fundação Amazônia Paraense de Amparo à Pesquisa (FAPESPA), Brasil; 3 Bolsista da Coordenação de Aperfeiçoamento de Pessoal de Nível Superior (CAPES), Brasil; 4 Universidade do Estado do Pará, Escola de Enfermagem Magalhães Barata, Belém, PA, Brasil; 5 Universidade do Estado do Pará, Departamento de Enfermagem Comunitária, Belém, PA, Brasil

**Keywords:** Letramento em Saúde, Qualidade de Vida, Fatores Socioeconômicos, Saúde da População Rural, Enfermagem Rural, Atenção Primária à Saúde

## Abstract

analisar o letramento funcional em saúde e a qualidade de vida relacionada à saúde em população ribeirinha usuária da atenção primária, segundo variáveis sociodemográficas.

estudo analítico, transversal, com 312 usuários de estratégia saúde da família ribeirinha. Os dados foram coletados por meio do teste de letramento em saúde, *12-item Health Survey* e um questionário socioeconômico adaptado pelos pesquisadores. Foi realizada correlação de Spearman, e testes de Mann-Whitney, Kruskal-Wallis e regressão logística múltipla, considerando p≤0,05.

apresentaram letramento funcional em saúde inadequado, 65,7%, e maior risco para homens (p<0,001), com idade entre 40–49 (p=0,010) e 50–59 anos (p=0,031), ensino fundamental incompleto (p<0,001) e completo (p=0,024), residindo longe do serviço de saúde (p<0,001). A qualidade de vida não mostrou associação ao letramento em saúde, entretanto, menor qualidade de vida apresentou relação com o sexo feminino (p=0,049), ensino fundamental incompleto (p=0,016), uso de celular com internet e rádio (p=0,013), idade avançada (p<0,001), aumento do número de filhos (p=0,002) e menor idade de início das atividades laborais (p<0,001).

o letramento funcional em saúde dos ribeirinhos é inadequado e não está associado à qualidade de vida, porém, ambos sofrem influências do perfil sociodemográfico de ribeirinhos.

## Destaques

**(1)** O letramento funcional em saúde dos ribeirinhos é inadequado. **(2)** Letramento em saúde não teve associação com a qualidade de vida.**(3)** O letramento em saúde foi associado aos aspectos sociodemográficos dos ribeirinhos. **(4)** A qualidade de vida foi relacionada ao perfil sociodemográfico dos ribeirinhos.

## Introdução

O Letramento Funcional em Saúde (LFS) diz respeito à capacidade cognitiva da pessoa para ler, interpretar e decodificar informações disponíveis e repassadas no serviço de saúde, importantes para tomada de decisão terapêutica e manutenção do autocuidado e do bem-estar^(1)^.

No desenvolvimento deste estudo adotaram-se as concepções do modelo teórico de Letramento em Saúde (LS)^(2)^ que considera o conhecimento, a motivação e as competências para manutenção ou melhora da Qualidade de Vida (QV). Trata-se de um modelo que abarca três grupos de fatores, do mais distal ao proximal, os quais são: determinantes socioambientais, envolvendo demografia, cultura e sistemas sociais; fatores situacionais que envolvem suporte familiar e mídia; e, fatores pessoais abarcando variáveis socioeconômicas.

A QV alcança conceito amplo com dimensão nas esferas: física; mental e social. Está diretamente ligada à capacidade funcional, ao bem-estar socioeconômico e ao grau de satisfação da pessoa. Os componentes físicos e mentais incorporam variáveis importantes para avaliar a Qualidade de Vida Relacionada à Saúde (QVRS)^(3)^.

Trata-se de uma temática que desperta interesse global, ratificado nas evidências científicas a partir da busca realizada em sete bases de dados, incluindo a *Medical Literature Analysis and Retrievel System Online*, identificando-se diversos estudos que abordavam o LS e QV, entretanto, somente cinco com população rural, e nenhum com ribeirinhos.

Dentre os estudos de associação do LS e QV evidenciaram-se repercussões positivas relacionadas à autopercepção do estado de saúde^(4-9)^, autogestão^(10-14)^, intervenções focadas em promoção à saúde^(15-16)^, adesão ao tratamento^(17)^, saúde física^(18-19)^ e saúde mental^(20-22)^. As repercussões negativas estiveram relacionadas à falta de acesso a informações sobre saúde, ao agravamento de condições clínicas e à maior procura por suporte médico^(23-24)^.

Destaca-se estudo realizado na Alemanha^(4)^, com pessoas atendidas em um sistema de saúde integrado, que guarda semelhança com o contexto da Atenção Primária à Saúde (APS), que confirmou associação entre LS adequado e elevada QVRS, em território de ruralidade. Em que pese as características geográficas de área rural, não se configuram ribeirinhos.

Outra iniciativa diz respeito ao estudo realizado na China^(20)^, com pessoas em estado de pré-diabetes, no contexto da APS, que identificou associação entre LS adequado e elevada QVRS. Embora tenha sido realizado com moradores de área rural, não se trata de comunidade ribeirinha.

No Brasil, estado do Pará, pesquisa abordando o LFS, revelou associação do letramento inadequado à baixa escolaridade e baixa renda, ratificando repercussão no déficit do autocuidado e da promoção da saúde de usuários da APS^(1)^ . Ressalta-se que o Pará integra a Amazônia brasileira, onde reside expressiva população ribeirinha, cujo contexto de vida é peculiar, podendo repercutir nos indicadores de morbimortalidade, requerendo observação atenta ao perfil sociodemográfico e ao modo de vida^(25)^.

Os ribeirinhos dependem quase exclusivamente do Sistema Único de Saúde (SUS) e procuram o serviço mais próximo de sua residência, porém, são condicionados a ambientes de difícil locomoção com precariedade de meios de transporte. A atuação das equipes de APS nessas comunidades precisa garantir resolutividade, entretanto, enfrenta desafios geográficos, quadro reduzido de profissionais com alta rotatividade em razão de vínculos trabalhistas frágeis^(26)^.

Há que se destacar que a atuação das equipes de APS e dos gestores em saúde necessita pautar-se na Política Nacional de Saúde Integral das Populações do Campo, da Floresta e das Águas^(27)^ e na Política Nacional de Atenção Básica (PNAB)^(28)^ de modo a atender às necessidades específicas dos moradores locais. Na rotina de trabalho, é desafiante e imprescindível que os profissionais de saúde utilizem vocabulário e materiais educativos compreensivos ao entendimento dessas populações, o que remete ao campo do LS^(29)^.

Desse modo, a abordagem deste estudo é de grande interesse para o avanço da ciência, apresenta evidências científicas de população vulnerabilizada com peculiaridade no modo de vida e com possibilidades de replicação em outros espaços geográficos que compartilham características semelhantes às ribeirinhas, especialmente na América Latina.

Para melhor conhecer a relação entre o LFS e a QVRS em comunidade ribeirinha da Amazônia, este estudo objetivou analisar o letramento funcional em saúde e a qualidade de vida relacionada à saúde em população ribeirinha usuária da atenção primária, segundo variáveis sociodemográficas.

## Método

### Delineamento do estudo

Estudo analítico e transversal, pautado no *Strengthening the Reporting of Observational Studies in Epidemiology* (STROBE)^(30)^ como diretriz para redação do manuscrito.

### Local e população do estudo

Realizado em área ribeirinha do município de Abaetetuba, estado do Pará, Brasil, localizado no Baixo Tocantins, com população de 158.188, conforme o censo de 2022^(31)^. A região ribeirinha abrange 72 localidades, com aproximadamente 42 mil habitantes^(32)^.

O estudo foi realizado em território adscrito da equipe Saúde da Família Ribeirinha (eSFR) que atende usuários de quatro localidades, denominadas neste estudo de rios: X, Y, Z, W. A eSFR está localizada no rio X e alcança oito microáreas, sendo duas sem cobertura de Agentes Comunitários de Saúde (ACS), pertencentes ao rio Z. A escolha das localidades deu-se por ser uma população assistida pela única eSFR da Região.

Conforme o cadastro no Sistema de Territorialização do Sistema Único de Saúde (e-SUS-território) o rio X possui 461 habitantes com idade ≥ 18 anos, o rio Y, 133, o rio Z, 118 e o rio W, 668, totalizando 1.380 usuários, atendidos pela eSFR. Em adição, no rio Z existem ainda moradores não quantificados por não possuírem cadastro no e-SUS-território em razão de residirem em áreas geográficas não assistidas por ACS.

### Critérios de seleção

Incluíram-se usuários com idade ≥ 18 anos, de ambos os sexos que sabiam ler e escrever, residentes na região ribeirinha do município de Abaetetuba, em território adscrito da eSFR. Excluíram-se pessoas com limitações visuais e/ou auditivas que impediam a leitura dos instrumentos ou mesmo ouvir o entrevistador por ocasião da coleta de dados, além de pessoas com hipossuficiência cognitiva ou comportamental que dificultasse a participação no estudo, previamente identificadas em registros no e-SUS-território e/ou autorreferidas pelos participantes ou familiares.

### Definição da amostra

A amostra foi constituída por 312 usuários, definida a partir da técnica de amostragem de Cochran^(33)^, considerando o tamanho da população e o erro amostral de 5%.

Optou-se por inserir no estudo representantes dos quatro rios, proporcionalmente, considerando o cadastro dos usuários no e-SUS-território. Consideraram-se, também, os diferentes estratos de escolarização, segundo dados divulgados pelo Instituto Brasileiro de Geografia e Estatística (IBGE), ao reconhecer as seguintes proporções: 38,6% da população correspondente a pessoas sem instrução e ensino fundamental incompleto; 12,5% com fundamental completo e ensino médio incompleto; e, 48,8% com ensino médio completo e mais^(34)^.

A amostragem foi não probabilística por cotas^(35)^, visto que a seleção dos participantes foi pela representação proporcional das quatro localidades, observando o quantitativo de pessoas residentes no território correspondentes aos três estratos de escolaridade. Essa opção possibilitou obtenção de dados representativos de cada rio.

A inserção das quatro localidades se deu para contemplar a diversidade populacional e a estratificação por escolaridade, evitando, assim, viés amostral capaz de repercutir nos níveis de letramento, uma vez que a escolarização vem exibindo relação com o LS^(29,36)^.

### Instrumentos utilizados e variáveis do estudo

Utilizou-se o Teste de Letramento em Saúde (TLS), validado no Brasil em 2019 e adaptado transculturalmente para a língua portuguesa do Brasil^(36-37)^, composto por duas partes: avaliação do domínio de numeramento com 17 itens e avaliação de leitura e compreensão com 50 itens. A opção pelo mesmo deu-se pela possibilidade de avaliar ambos os domínios. A consistência interna foi de 0,953 e sua aplicação seguiu as recomendações dos autores^(36-37)^.

Os escores atribuídos ao TLS seguiram as recomendações do instrumento original^(37)^. Para seu cálculo atribuiu-se pontuação um para cada resposta correta e zero para respostas incorretas e/ou autodeclaração de não saber e/ou não preenchimento. Em relação à parte numérica, a pontuação atribuída, até 17 no escore bruto, foi multiplicada por 2,941, a fim de converter para pontuação de zero a 50^(37)^.

Na parte da leitura não houve ponderação. Ao final, calcularam-se os escores pela somatória dos domínios, sendo classificado como letramento adequado os que alcançaram entre 75 e 100 pontos, letramento limitado os que obtiveram entre 60 e 74 e letramento inadequado os que ficaram na faixa de zero a 59 pontos^(36)^.

Para medir a QVRS foi utilizado o *12-itemHealth Survey* (SF-12), versão 1.0, criado em 1994^(38)^ e validado no Brasil em 2004^(39)^, constituído por 12 itens, e dividido em dois componentes, o físico, denominado *Physical Component Summary*(PCS), e o mental, definido como *Mental Component Summary*(MCS). Cada componente apresenta quatro domínios: o PCS envolve a saúde geral, dor, capacidade funcional e aspecto físico; e, o MCS contempla os domínios de aspecto emocional, saúde mental, vitalidade, além do aspecto social^(39)^.

A opção pelo SF-12 se deu por ser um instrumento utilizado para avaliação de saúde geral, de formato simples, curto, com rápida aplicação, amplamente usado para medir com precisão e eficiência a saúde física e mental em pesquisa na população geral. Apresenta alto grau de confiabilidade, obtendo coeficiente de alfa de Cronbach igual a 0.836^(40-41)^.

O cálculo dos escores do SF-12 seguiu as recomendações do instrumento original^(38)^, que apresenta escala graduada, do tipo Likert, cuja pontuação varia conforme a composição da pergunta, com opção de duas a seis respostas, oscilando de zero a 100, sendo os maiores escores associados a melhores níveis de QV. A análise de cada componente foi feita isoladamente, não estabelecendo escore total^(38-39)^, porém foram agrupados em PCS e MCS. Para a definição do escore dos componentes foi considerado o algoritmo de cálculo da pontuação, considerando-se a média da população norte-americana de 1998, que vem sendo adotada em estudos brasileiros e internacionais^(38-41)^.

Para traçar o perfil sociodemográfico, foi utilizado um questionário elaborado pelo núcleo de pesquisa e confederação nacional dos trabalhadores e trabalhadoras na agricultura, estruturado para execução do projeto “Escuta itinerante: acesso das populações do campo e da floresta ao SUS”^(42)^. O instrumento apresenta duas partes: a primeira diz respeito ao perfil e modo de vida; produção e trabalho no campo; e, sistema de saúde. A segunda parte corresponde à identidade de sexo e idade dos participantes.

Deste instrumento, foram extraídas sete questões que guardam relação com o presente estudo e permitiram obter os dados relativos ao perfil sociodemográfico dos ribeirinhos. Tais questões estão contempladas na primeira parte do questionário, sendo seis referentes ao perfil/modo de vida, que inclui sexo, idade, localidade, escolaridade, número de filhos e meios de comunicação. A outra questão diz respeito à produção/trabalho no campo, que permitiu investigar a idade de início das atividades laborais. Tais variáveis foram adaptadas à luz de pesquisas realizadas com população ribeirinha da Amazônia^(3,43)^.

Realizou-se estudo**-**piloto com ribeirinhos usuários da APS, residentes em outras localidades do município, o que permitiu adequar os termos do TLS à cultura local considerando o sentido semântico. Foi importante, ainda, para direcionar o treinamento dos entrevistadores e planejar o trabalho de campo.

As adequações realizadas no TLS são referentes à inserção de alguns termos explicativos para palavras técnicas, visando facilitar o entendimento dos participantes. No teste de numeramento, foram inseridas palavras para explicar os seguintes termos: cápsula = pílula; antitérmico = remédio para tratar febre; e tylenol = paracetamol. No teste de compreensão de leitura, foram acrescentadas as seguintes explicações: pessoa que tem asma = asmático; remédio para a azia = antiácido; doença de osso = osteoporose; baixo açúcar no sangue = hipoglicemia; e doença mental = esquizofrenia.

### Coleta de dados

A coleta de dados ocorreu entre fevereiro e maio de 2021, no prédio da eSFR, enquanto aguardavam atendimento e nas visitas domiciliares programadas pelos ACS, a critério dos participantes. O tempo médio de duração das entrevistas foi de vinte e três minutos.

O TLS foi autopreenchido na parte de compreensão e leitura, na qual o entrevistado preenchia as lacunas selecionando a possibilidade mais apropriada para o texto, avaliada pelos trechos: A que continha instruções para o procedimento de radiografia, trechos B contemplava o texto inicial da legislação brasileira acerca do SUS e trecho C que consistia em um termo de consentimento de procedimentos. A parte de numeramento contou com auxílio do entrevistador, que disponibilizou os cartões com informações numéricas, e na sequência realizou o questionamento previsto no instrumento. Foi contabilizado o tempo de aplicação de cada parte do TLS^(36)^. O SF-12 foi aplicado com o auxílio do entrevistador.

Os entrevistadores foram a pesquisadora principal e cinco acadêmicos do Curso de Graduação em Enfermagem de uma universidade pública estadual, que cursavam a disciplina Enfermagem e as Populações Tradicionais da Amazônia, previamente treinados e supervisionados pela pesquisadora principal.

Os obstáculos vivenciados durante a coleta de dados envolveram limitações logísticas, a exemplo de: navegação em rios pequenos assoreados e na presença de maresias, passagem por furos, dificuldade para alocação de transporte seguro para a equipe e horário conflitante da coleta de dados com o turno das atividades laborais dos ribeirinhos.

Para superação dos obstáculos, a equipe de pesquisa contou com o apoio de lideranças comunitárias que abrigaram os pesquisadores em suas residências, viabilizando permanência no território, o que proporcionou melhor aproveitamento do tempo de coleta de dados. A colaboração repercutiu ainda na contratação de embarcação de médio porte com capacidade para enfrentamento de maresias.

### Tratamento e análise dos dados

Inseriram-se dados com dupla entrada em banco no *Statistical Package for the Social Sciences*, versão 21. A correlação de Spearman foi eleita para comparação entre pontuações, escores e idades. Para avaliar a normalidade utilizou-se o teste de Shapiro-Wilk. Na análise bivariada desses escores com as variáveis sociodemográficas, aplicou-se os testes de Mann-Whitney e Kruskal-Wallis, que permitiu comparar os escores com as variáveis categóricas.

O modelo de regressão logística foi aplicado para medir a associação entre as variáveis de interesse (sexo, idade, localidade, escolaridade, números de filhos, meios de comunicação e início de trabalho laboral) e o desfecho LFS inadequado. As variáveis que tiveram associação significativa na análise de regressão logística bivariada permaneceram no modelo ajustado. Todas as análises foram realizadas no pacote estatístico R, versão 4.1.1, considerando nível de significância de 0,05.

### Aspectos éticos

O estudo seguiu as Resoluções 466/12 e 580/18 do Conselho Nacional de Saúde/Ministério da Saúde, e aprovado pelo Comitê de Ética em Pesquisa do Curso de Graduação em Enfermagem da Universidade do Estado do Pará, sob o parecer 4.517.829 de 01 de fevereiro de 2021.

## Resultados

Dos 312 ribeirinhos que compuseram a amostra, 67,0% (n=209) eram do sexo feminino e a maioria tinha de dois a três filhos, com média de 2,18±1,999, variando de zero a 13 filhos. A média de idade atingiu 35,03±13,397 anos, variando de 18 a 78 anos, tendo maior frequência (71,5%) de idade entre 18 e 39 anos. Do total da amostra, 61,5% usavam telefone celular (Tabela 1).

A média de início da vida laborativa foi 12,51±4,518 anos e variação de 3 a 31 anos, sendo que metade da amostra iniciou atividades na faixa etária de 3 a 13 anos (n=156; 50,0%). A média do escore total do TLS foi 52,235±21,638, com variação de 5,941-99,997. Do total, 205 (65,7%) apresentaram LFS inadequado, 54 (17,3%) limitado e 53 (17,0%) adequado. A pontuação média do LFS total (p<0,001) apresentou redução proporcional ao comprometimento do letramento. Foram identificadas maiores médias de LFS no sexo feminino (p<0,001), local de residência no rio X (p<0,001) e o uso de telefone celular com internet (p<0,001). Menores médias foram identificadas entre residentes do rio W (p<0,001) sem acesso a aparelhos de meios de comunicação (p<0,001). O LFS reduziu com o aumento do número de filhos (p<0,001) e da idade (p<0,001), aumentou com a elevação da escolaridade (p<0,001) e idade de início das atividades laborais (p<0,001) (Tabela 1).

**Tabela 1 -:** Análise do letramento funcional em saúde segundo variáveis sociodemográficas dos ribeirinhos (n* = 312). Abaetetuba, PA, Brasil, 2021

			**Escore do TLS** ^†^		
**Variável**	**Categoria**	n* (%) ^‡^	**Média**	**Erro-padrão**	**p-valor** ^§^
**Sexo**					
	Feminino	209 (67,0%)	55,530	1,531	<0,001
	Masculino	103 (33,0%)	45,551	1,873	
**Número de filhos**					
	0	61 (19,6%)	59,157	2,723	<0,001
	1	66 (21,2%)	56,756	2,353	
	2 a 3	123 (39,4%)	51,940	1,997	
	4 a 13	58 (18,6%)	40,563	2,540	
	NI ^||^	4 (1,3%)	50,410	10,754	
**Idade (anos)**					
	18 a 29	122 (39,1%)	59,462	1,816	<0,001
	30 a 39	101 (32,4%)	53,331	2,178	
	40 a 49	37 (11,9%)	44,906	3,079	
	50 a 59	24 (7,7%)	38,150	4,291	
	60 a 78	24 (7,7%)	35,775	3,078	
	NI ^||^	4 (1,3%)	55,190	10,176	
**Localidade**					
	Rio X	111 (35,6%)	59,276	2,118	<0,001
	Rio Y	33 (10,6%)	49,232	3,282	
	Rio Z	29 (9,3%)	53,783	3,725	
	Rio W	139 (44,6%)	47,003	1,747	
**Escolaridade**					
	EFI ^¶^	132 (42,3%)	37,432	1,383	<0,001
	EFC**	32 (10,3%)	53,138	3,135	
	EMI ^††^	30 (9,6%)	56,120	3,164	
	EMC ^‡‡^	85 (27,2%)	64,998	1,854	
	ESI ^§§^	15 (4,8%)	78,978	4,232	
	ESC ^||||^	18 (5,8%)	70,158	4,443	
**Comunicação**					
	Telefone celular	192 (61,5%)	46,839	1,434	<0,001
	Celular e internet	110 (35,3%)	62,982	1,995	
	Celular, internet e rádio	3 (1,0%)	44,743	9,127	
	Não possui	3 (1,0%)	39,508	7,172	
	NI ^||^	4 (1,3%)	30,881	8,662	
**Início de trabalho**					
	3 a 13 anos	156 (50,0%)	45,240	1,544	<0,001
	14 a 17 anos	60 (19,2%)	52,62	2,629	
	18 a 31 anos	39 (12,5%)	67,310	3,038	
	Nunca trabalhou	55 (17,6%)	61,030	3,081	
	NI ^||^	2 (0,6%)	50,440	20,794	

*n = Número de participantes; ^†^TLS = Teste de Letramento em Saúde; ^‡^% = Frequência percentual; ^§^p-valor = Teste de Mann-Whitman para variáveis com duas categorias e de Kruskal-Wallis para as demais, significativo para p≤0,05; ^||^NI = Não Informado; ^¶^EFI = Ensino Fundamental Incompleto; **EFC = Ensino Fundamental Completo; ^††^EMI = Ensino Médio Incompleto; ^‡‡^EMC = Ensino Médio Completo; ^§§^ESI = Ensino Superior Incompleto; ^||||^ESC = Ensino Superior Completo

Em relação à QVRS, o PCS e MCS apresentaram média de 43,461±10,176 e 47,752±9,633, com mediana de 44,730 e 50,191, tendo variação de 14,063-62,075 e 19,317-66,982, respectivamente. O PCS (p=0,090) e MCS (p=0,776) não mostraram associação ao LFS, entretanto, o sexo feminino (p=0,049) apresentou diferença estatística com menor QVRS no MCS (Tabela 2).

Em relação ao PCS, identificou-se menor QVRS nos participantes com ensino fundamental incompleto (p=0,016), que usavam celular com internet e rádio (p=0,013). Entretanto, quem não utilizava aparelho de meio de comunicação apresentou melhor QV no PCS (p=0,013). O PCS da QVRS reduziu com o aumento do número de filhos (p=0,002), o avanço da idade até 59 anos (p<0,001) e a menor idade de início das atividades laborais (p<0,001) (Tabela 2).

**Tabela 2 -:** Análise da qualidade de vida relacionada à saúde segundo o letramento funcional em saúde e características sociodemográficas dos ribeirinhos (n* = 312). Abaetetuba, PA, Brasil, 2021

		**PCS** ^†^				**MCS** ^‡^	
**Variável**	**Categoria**	**Média**	**Erro-padrão**	**p-valor** ^§^	**Média**	**Erro-padrão**	**p-valor** ^§^
**LFS** ^||^							
	Inadequado	42,495	0,749	0,090	47,744	0,667	0,776
	Limitado	45,724	1,246		48,239	1,424	
	Adequado	44,892	1,161		47,285	1,267	
**Sexo**							
	Feminino	43,346	0,737	0,813	47,042	0,655	0,049
	Masculino	43,695	0,903		49,192	0,970	
**Número de filhos**							
	0	46,212	1,044	0,002	46,674	1,216	0,084
	1	46,235	1,161		49,644	1,237	
	2 a 3	42,218	0,949		48,063	0,859	
	4 a 13	40,145	1,446		46,603	1,218	
	Não informado	42,046	4,062		40,040	4,766	
**Idade (anos)**							
	18 a 29	46,197	0,835	<0,001	48,121	0,887	0,180
	30 a 39	44,823	0,919		47,779	0,917	
	40 a 49	39,204	1,767		46,036	1,562	
	50 a 59	34,553	2,197		50,625	1,844	
	60 a 78	39,388	1,935		44,526	2,275	
	Não informado	42,899	5,886		53,780	2,088	
**Escolaridade**							
	EFI ^¶^	41,241	0,901	0,016	47,605	0,845	0,364
	EFC**	43,790	2,066		45,994	1,620	
	EMI ^††^	46,978	1,657		48,250	1,488	
	EMC ^‡‡^	44,883	0,998		48,710	1,099	
	ESI ^§§^	46,860	2,287		44,267	2,590	
	ESC ^||||^	43,750	2,526		49,504	2,240	
**Comunicação**							
	Telefone celular	42,006	0,763	0,013	47,991	0,697	0,393
	Celular e internet	45,876	0,853		47,874	0,904	
	Celular, internet e rádio	37,264	5,894		38,000	7,060	
	Não possui	54,009	3,015		43,177	5,167	
	Não informado	43,647	5,366		43,650	5,578	
**Início de trabalho**							
	3 a 13 anos	40,964	0,877	<0,001	47,468	0,797	0,816
	14 a 17 anos	44,943	1,178		47,189	1,317	
	18 a 31 anos	45,213	1,473		49,666	1,389	
	Nunca trabalhou	47,850	1,032		47,881	1,157	
	Não informado	38,956	1,476		45,914	13,167	

*n = Número de participantes; ^†^PCS = *Physical Component Summary;* ^‡^MCS = *Mental Component Summary*; ^§^p-valor = Teste de Mann-Whitman para variáveis com duas categorias e de Kruskal-Wallis para as demais, significativo para p≤0,05; ^||^LFS = Letramento Funcional em Saúde; ^¶^EFI = Ensino Fundamental Incompleto; **EFC = Ensino Fundamental Completo; ^††^EMI = Ensino Médio Incompleto; ^‡‡^EMC = Ensino Médio Completo; ^§§^ESI = Ensino Superior Incompleto; ^||||^ESC = Ensino Superior Completo

A Tabela 3 indica que quanto melhor o PCS maior a habilidade de leitura (p≤0,01), maior a pontuação no trecho A (p≤0,05) e B (p≤0,05) da leitura. Entretanto, quanto pior o PCS, maior a idade (p≤0,01), e mais tempo usado no preenchimento do trecho A (p≤0,05) e do total dos trechos de leitura (p≤0,05). Não houve correlação significativa entre os MCS e os itens de mensuração da TLS.

**Tabela 3 -:** Correlação de Spearman entre o componente físico e mental da qualidade de vida relacionada à saúde com idade e teste de letramento em saúde dos ribeirinhos (n* = 312). Abaetetuba, PA, Brasil, 2021

**Variáveis**	**PCS** ^†^	**MCS** ^‡^
Idade	-0,334 ^§^	-0,059
Tempo trecho A da leitura	-0,172 ^||^	-0,019
Tempo trecho B da leitura	0,053	0,021
Tempo trecho C da leitura	0,09	0,011
Tempo total de leitura	-0,152 ^||^	-0,025
Escore do trecho A da leitura	0,159 ^||^	0,055
Escore do trecho B da leitura	0,150 ^||^	0,065
Escore do trecho C da leitura	0,142	-0,001
Escore total de leitura	0,189 ^§^	0,073
Escore de numeramento	0,06	0,082
Tempo do teste de numeramento	-0,115	0,017
Escore total do teste de letramento em saúde	0,140	0,084

*n = Número de participantes; ^†^PCS = *Physical Component Summary*; ^‡^MCS = *Mental Component Summary*; ^§^≤0,01; ^||^>0,01 - ≤0,05

Os modelos bivariados e ajustados estão apresentados na Tabela 4. O ajuste do modelo indica que o uso de telefone celular com internet pode ser um fator de proteção para o LFS inadequado. Ademais, pessoas do sexo masculino, idade na faixa de 40 a 49 e 50 a 59, mais distante do serviço de saúde (Rio W), ter cursado até ensino fundamental (incompleto e completo) aumentaram o risco de LFS inadequado.

**Tabela 4 -:** Associação do letramento inadequado com as características sociodemográficas (n* = 312). Abaetetuba, PA, Brasil, 2021

	**Modelo bivariado**		**Modelo ajustado**	
**Características**	**OR** ^†^ **(I** C **95%)** ^‡^	**p-valor** ^§^	**OR** ^†^ **(I** C **95%)** ^‡^	**p-valor** ^§^
Sexo (referência = Feminino)				
Masculino	2,96 (1,72; 5,29)	<0,001	4,42 (2,11; 9,73)	<0,001
Número de filhos (referência=0 filhos)				
1 filho	1,32 (0,66; 2,66)	0,438		
2 ou 3 filhos	2,5 (1,33; 4,74)	0,005		
4 filhos ou mais	4,79 (2,16; 11,3)	<0,001		
Idade (referência=18 a 29 anos)				
30 a 39 anos	1,75 (1,02; 3,01)	0,043	1,51 (0,72; 3,20)	0,278
40 a 49 anos	7,98 (2,95; 28,0)	<0,001	6,01 (1,68; 26,4)	0,010
50 a 59 anos	6,77 (2,19; 29,7)	0,003	5,92 (1,29; 34,6)	0,031
60 anos ou mais	10,6 (2,96; 68,3)	0,002	3,66 (0,67; 31,9)	0,174
Não informado	0,97 (0,11; 8,28)	0,974	0,94 (0,05; 14,3)	0,963
Localidade (referência=Rio X)				
Rio Y	2,18 (0,97; 5,19)	0,066	1,81 (0,61; 5,62)	0,290
Rio Z	1,55 (0,68; 3,67)	0,305	1,6 (0,45; 5,74)	0,468
Rio W	3,17 (1,85; 5,50)	<0,001	3,7 (1,78; 7,94)	<0,001
Escolaridade (referência=Superior completo)				
Superior incompleto	0,31 (0,04; 1,64)	0,195	0,43 (0,05; 2,91)	0,410
Fundamental completo	5,11 (1,53; 18,9)	0,010	5,61 (1,31; 26,8)	0,024
Fundamental Incompleto	31 (9,67; 112)	<0,001	16,5 (4,35; 70,3)	<0,001
Médio completo	1,21 (0,42; 3,76)	0,731	1,03 (0,29; 3,99)	0,959
Médio Incompleto	3 (0,91; 10,8)	0,078	2,91 (0,69; 13,4)	0,156
Meios de comunicação (referência=Telefone celular)				
Telefone celular e internet	0,25 (0,15; 0,40)	<0,001	0,47 (0,23; 0,94)	0,033
Telefone celular, internet e radio	0,61 (0,06; 13,4)	0,692	2,47 (0,09; 149)	0,645
Não possui	4791233 (0,00; Inf ^||^ )	0,986	3900155 (0,00; Inf ^||^ )	0,985
Início do Trabalho (referência=3 a 13 anos)				
14 a 17 anos	0,6 (0,31; 1,20)	0,141		
18 a 31 anos	0,13 (0,06; 0,27)	<0,001		
Nunca trabalhou	0,22 (0,11; 0,41)	<0,001		

*n = Número de participantes; ^†^OR = *Odds Ratio;* ^‡^IC = Intervalo de confiança (95%); ^§^p-valor = Teste de Mann-Whitman para variáveis com duas categorias e de Kruskal-Wallis para as demais, significativo para p≤0,05; ^||^Inf = Infinito

## Discussão

O LFS mostrou-se inadequado na maioria dos participantes. Nesse sentido, estudo realizado no Brasil^(36)^, que validou o TLS, identificou que, aproximadamente, metade dos 302 participantes apresentavam LFS inadequado ou limitado, justificado por questões sociodemográficas, sobretudo pela elevação da idade e redução dos anos de estudos.

Em contraste, pesquisa realizada em áreas rurais da Alemanha concluiu que 61,9% tinham LS suficiente. Embora a amostra tenha sido composta de extratos populacionais com perfil social e educacional desfavoráveis, o nível de LS foi explicado pelo investimento em prevenção, promoção à saúde e empoderamento da gestão, produzindo efeito positivo nas pessoas^(4)^.

À luz dessas discussões e pelo perfil sociodemográfico dos ribeirinhos, justifica-se a prevalência de inadequada habilidade básica de numeramento e compreensão de leitura, necessários para entender informações de saúde, sendo que a distância e limitações logísticas para acessar serviços de saúde podem interferir no desempenho do LFS. Ademais, as vulnerabilidades sociais presentes no contexto local podem impactar no uso de informações prestadas pelo serviço de saúde e repercutir nas decisões individuais.

Nesse sentido, a maior proporção de mulheres, foi, da mesma forma, divulgada na literatura nacional e internacional de LS, indicando predomínio do sexo feminino^(1,15,36,44-45)^, podendo-se atribuir-se ao fato de as mulheres procurarem mais os serviços de saúde e à ausência dos homens nos ambientes familiares durante a coleta de dados devido às atividades laborais.

É imputado à mulher ribeirinha da Amazônia desempenhar importante elo da rede de apoio e cuidado familiar, acumulando atividades do lar sem compensação de renda, com perfil de baixa escolaridade, gravidez na adolescência, elevado índice gestacional e de fecundidade. Esses resultados podem orientar a construção de projetos terapêuticos relevantes no contexto do LS de mulheres ribeirinhas da Amazônia^(43)^.

Em relação ao LFS, as mulheres apresentaram melhores níveis de habilidade de numeramento e compreensão de leitura e menor risco de LFS inadequado, condizente com estudo realizado no Japão^(45)^, no qual as mulheres apresentaram melhor habilidade do LS, para tomada de decisões. Embora o estudo tenha sido produzido em outro país, visualiza-se semelhança com o contexto ribeirinho da Amazônia paraense, em que a mulher assume condição de principal cuidadora na família, sendo mais propensa a aplicar medidas de promoção da saúde, além do papel de gerenciamento de consultas e medicamentos e maior presença nos serviços de saúde.

Por outro lado, as mulheres apresentaram maior comprometimento QV mental, ressaltando-se a dimensão do universo feminino que envolve atividades peculiares do modelo patriarcal, no qual cabe à mulher o cuidado com os filhos e o lar, situações que impactam na estabilidade emocional e psicológica, capazes de desencadear, inclusive, quadros depressivos^(46)^.

Acerca da fecundidade, identificou-se que a média de filhos é equivalente ao valor de reposição populacional de 2,1 filhos por mulher, superando as cifras registradas no Brasil, correspondente a 1,4 filhos por mulher, segundo pesquisa da Organização das Nações Unidas, que admitiu maior taxa de fecundidade entre pessoas com menor escolarização, mais pobres, oriundas de áreas rurais e da região norte do Brasil^(47)^.

Constatou-se relação inversa entre LFS e o número de filhos, pois quanto menor o número de filhos, melhor o LFS e aqueles que não tinham filhos alcançaram LFS ainda maior. Nesse sentido, inquérito de saúde conduzido em comunidades ribeirinhas do Amazonas^(43)^ detectou que as mulheres tinham as primeiras gestações ainda na adolescência e elevada fecundidade, devendo ser considerado para adoção de medidas de educação reprodutiva sob o aporte teórico do LS e assim alcançar melhor QV.

A avaliação da QV física permitiu identificar associação com o número de filhos devido às exigências do ato de cuidar em conjunto com atividades laborais e execução das tarefas domésticas que requerem esforços físicos. Nesse sentido, estudo de revisão sistemática referiu influência negativa do número de filhos na QV de cuidadores familiares quando relacionada ao aspecto emocional e vitalidade^(48)^.

Em relação às peculiaridades da idade, constatou-se que a frequência de participantes reduziu com o aumento da idade, havendo maior proporção de participantes adultos jovens, aproximando-se da média de idade obtida em estudo de LS e QV, realizado na zona rural e urbana da China^(49)^. Além disso, evidenciou-se a relação inversa entre idade e LS, ou seja, quanto mais avançada a idade, pior o LS e maior risco de LFS inadequado, convergindo com investigação realizada no Brasil acerca da influência do LS em pacientes com doença arterial coronariana^(50)^.

Esse cenário pode ser decorrente de limitações inerentes ao processo de envelhecimento, que pode ocasionar o comprometimento da capacidade de assimilação das informações, influenciada por variáveis como anos de estudo e hábitos de leitura. À medida que a idade aumenta, a capacidade cognitiva é afetada, diminuindo a capacidade de entender sobre conhecimentos de saúde^(45,51)^, resultante da limitação no entendimento dos quadros de adoecimento e nas formas de trabalho dos ribeirinhos, com: baixa escolaridade, exposição a riscos ergonômicos resultantes de atividades que exigem grande esforço físico, postura inadequada, jornada de trabalho prolongada, monotonia, repetitividade e imposição de rotina intensa.

A inexistência de escolas em algumas localidades e a restrição de acesso ao ensino superior público podem potencializar o limitado conhecimento em relação à saúde, considerado o resquício histórico da educação precária e menos acessível, principalmente, para pessoas vulnerabilizadas^(52)^.

Deste modo, a escolaridade está diretamente relacionada ao LS, isto é, quanto mais elevada, melhor o LS. Menor escolarização apresenta maiores riscos de LFS inadequado e tem relação com o nível de desenvolvimento do país, tendo em vista que quanto maior o desenvolvimento, melhores os índices de escolarização e LS^(53)^.

Neste estudo, pessoas que residiam mais distantes do serviço de APS apresentaram pior LFS e maior risco de LFS inadequado quando comparado com aquelas que residem na localidade na qual o serviço está implantado, atribuível ao difícil acesso ao serviço e, consequentemente, menor procura pelo atendimento. Portanto, frequentar os serviços de saúde impacta no nível de LFS dessas populações^(54)^ e o acesso limitado a informações vem sendo associado às condições e contextos desfavoráveis em saúde^(55)^.

A localidade mais distante, embora apresente cobertura pelo ACS, é uma região com reconhecida presença de maresias e difícil deslocamento pelo tráfego intenso de embarcações no trajeto para eSFR. As populações ribeirinhas têm limitações para acessar o serviço de saúde devido às peculiaridades de acessibilidade e aos desafios geográficos, regidos pela presença de maresias, ausência de transportes fluviais e dinâmicas das marés^(32)^. Essa realidade contribui para a exclusão desses povos das ações e serviços de saúde e remetem à necessidade da atuação da APS para melhorar o nível de LS e QV^(26)^.

Há que se valorizar o processo saúde-doença do ribeirinho compreendido no contexto da diversidade cultural e do modo de vida^(29)^. Nesse sentido, é imprescindível que a APS se aproprie das especificidades e da cultura local para promover o LS das pessoas, com informações relevantes e reconhecidas como significativas à sua realidade, que favoreça o bem-estar e a adesão a tratamentos e, consequentemente, promova a QV.

Nas peculiaridades da vida ribeirinha, constatou-se que o meio de comunicação mais utilizado é o telefone móvel com ou sem internet, condizente com a pesquisa do IBGE, que em 2019, confirmou a existência de telefone móvel em 94,0% dos domicílios do país. Há que se esclarecer que há restrições para o uso da internet em áreas rurais, sobretudo pela dificuldade de aquisição de aparelho compatível em razão do elevado custo e indisponibilidade do sinal de internet na área do domicílio^(56)^.

Ademais, muitas comunidades ribeirinhas, por não possuírem energia elétrica estável, limitam o acesso a informações^(25)^. O acesso aos meios de comunicação de maior desenvolvimento tecnológico favoreceu o LFS dos ribeirinhos, sendo considerado fator de proteção para o LFS inadequado, pois os que usavam celular com internet tiveram melhor LFS do que aqueles que não tinham aparelho ou usavam somente o celular.

Em decorrência do acelerado desenvolvimento da tecnologia de comunicação eletrônica e da gama de informações vinculadas a ela, é necessário melhorar o LS digital ou *eHealth literacy* e assim instrumentalizar os ribeirinhos para acessarem e usarem informações relevantes sobre saúde por meio da mídia eletrônica^(15)^.

A QV física mostrou-se mais comprometida entre aqueles que usavam rádio e celular com internet e menos entre os que não tinham aparelho de meio de comunicação. Depreende-se disso que o avanço da tecnologia de informação em regiões rurais, atrelado ao uso desmedido, gera dependência tecnológica e pode produzir inatividade física. Essa situação pode ser intensificada pelos reduzidos ambientes de interação e espaço de lazer no território, além do distanciamento físico produzido pela pandemia da COVID-19, fatores de interferência na saúde física.

É fato que a transformação advinda da internet reflete na redução da convivência na coletividade, porém, possibilita a incorporação de tecnologias de cuidado, a exemplo da telessaúde e teleconsultas. Estas encurtam distâncias geográficas, contribuindo para a efetividade e a resolutividade da assistência, principalmente, em áreas de difícil acesso ao serviço de saúde, como nas comunidades ribeirinhas da Amazônia, podendo melhorar os níveis de LS e impactando positivamente na QV física^(57)^.

Ainda no contexto de vida ribeirinho destaca-se o trabalho precoce de crianças e adolescentes decorrentes da negação e naturalização da exploração do trabalho infantil sob a égide do aumento da renda familiar e da oportunidade para o aprendizado no campo da prática^(58)^. Não obstante, as medidas governamentais são distribuídas contraditoriamente em relação aos níveis de trabalho infantil nos estados brasileiros, as inspeções são insuficientes e a política de transferência de renda exibe limitações no combate ao trabalho infantil^(59)^.

O trabalho infantil foi relacionado à menor QV no PCS, decorrente da exposição em atividades de grande esforço físico, tais como: agricultura de subsistência e pesca artesanal. Tal exposição pode produzir consequências sobre a saúde física, na medida que envolvem desordens do sistema musculoesquelético e acidentes de trabalho, repercutindo na QV^(60)^.

Ao analisar a idade de início das atividades trabalhistas, constatou-se relação direta com o LFS, destacando-se que crianças e adolescentes inseridos precocemente no mundo do trabalho exibiram pior LS quando comparados com aqueles que iniciaram na fase de aprendiz ou a partir de 18 anos. Isto se deve ao comprometimento intelectual que se expressa no baixo conhecimento, baixa motivação e reduzidas competências numéricas e de leitura, levando à evasão escolar e ao comprometimento da tomada de decisão em saúde^(61)^.

O LS é identificado como área-chave de ação para alcançar a agenda do desenvolvimento sustentável de 2030, contemplado no oitavo objetivo de desenvolvimento sustentável ao se reportar a necessidade de melhores condições de trabalho e crescimento econômico. Para tanto, melhores níveis de LS instrumentalizam trabalhadores rurais para a reivindicação de oportunidades e melhores condições de trabalho^(61)^.

No que concerne às oportunidades de trabalho e renda, estão atreladas à água e à floresta que constituem meio de subsistência. Eles vivem da agricultura familiar e pesca artesanal que agregadas à cultura configuram sua subjetividade. Em que pese a lógica capitalista considerar insuficiente e insustentável, a pesca e agricultura produzem soberania alimentar e abastecimento do comércio regional^(25)^.

Nesta investigação, os componentes da QVRS não se mostraram associados, significativamente, aos diferentes níveis de LFS, o que foi identificado em outros estudos que abordaram LS e QV, a saber: estudo que mediu o impacto do LS em pacientes submetidos à terapia renal substitutiva, associado aos aspectos cognitivos, adesão medicamentosa e QV^(62)^; estudo realizado com pessoas vulnerabilizadas que examinou o LS no controle do diabetes em coreano-americanos^(10)^; estudo que avaliou a relação do LS com o medo das pessoas relacionado a *Coronavirus Disease* 2019 (COVID-19)^(63)^; e, estudo que examinou a relação entre o LS, estilos de vida promotores de saúde e QV entre estudantes universitários^(64)^.

Por outro lado, iniciativas internacionais identificaram essa relação, conforme estudo que mediu as inter-relações entre demografia, LS, autopercepção do estado de saúde e QV de imigrantes coreanos e vietnamitas^(5)^; estudo que correlacionou LS com QV e estado de saúde em comunidades rurais da Alemanha^(4)^ e pesquisa que analisou QVRS de adolescentes e seus pais com LS no contexto da COVID-19^(65)^.

O retrato sociodemográfico dos ribeirinhos revela a necessidade de mapeamento do LS para otimizar o conhecimento e a comunicação em saúde, além de identificação das dificuldades de entendimento acerca das informações recebidas nos serviços de APS. Dessa forma, é importante que o serviço reconheça o LS como determinante social de saúde necessário para compreensão das condições sociodemográficas e do modo de vida das pessoas.

Admite-se que a investigação do LFS com pessoas vulnerabilizadas, ainda é pouco descrita pela literatura nacional e internacional, limitando a comparação dos resultados. Outra limitação refere-se ao instrumento de coleta de dados que contempla textos de autopreenchimento estranhos à realidade ribeirinha, podendo ter ocasionado viés de preenchimento.

Contudo, a contribuição deste estudo diz respeito à oferta de subsídios para o planejamento locorregional em saúde e das ações educativas da APS, sobretudo para a enfermagem, visando à implementação de práticas compatíveis com os níveis de LFS dos usuários, possibilitando elevar a QV.

A pesquisa poderá contribuir para promover intervenções no serviço, bem como incentivar a inserção de abordagem do LS nos currículos de graduação e pós-graduação em saúde. Poderá, ainda, favorecer potencial avanço na Política Nacional de Saúde Integral das Populações do Campo, da Floresta e das Águas, ao incorporar a perspectiva da intersetorialidade para promoção do LS e QV, articulando saberes tradicionais, políticas públicas de saúde e de educação. Assim, vislumbra-se oferta equânime e resolutiva de ações de educação em saúde, por meio de estratégias adequadas ao modo de vida das pessoas.

## Conclusão

Este estudo pioneiro no Brasil, que avaliou o LFS e QVRS de ribeirinhos usuários da APS na modalidade ribeirinha, evidenciou que não houve associação entre o LFS e PCS e MCS da QVRS, sendo possível identificar congruência do LFS e da QVRS com aspectos sociodemográficos.

Os ribeirinhos da APS apresentaram LFS inadequado, remetendo à necessidade de efetivação/implementação de política pública e planejamento estratégico específico para oferta de informações em saúde significativas e adequadas ao perfil sociodemográfico dessas populações, a fim de impactar na autogestão em saúde.

O retrato sociodemográfico dos ribeirinhos revela que homens, idosos, residentes mais distantes do serviço, sem acesso à internet, com menor escolarização e que sofreram exploração do trabalho infantil apresentaram pior LFS, requerendo da APS uma comunicação em saúde simples, objetiva e significativa ao seu modo de vida.

Para as intervenções de melhoria na QV dos ribeirinhos é necessário considerar que seu perfil retrata QV física com maior comprometimento em idosos, de baixa escolaridade, com acesso a meios de comunicação, em maior taxa de fecundidade e exploração de trabalho infantil, além de mulheres que tiveram pior QV mental.

De forma geral, este estudo mostrou que as principais variáveis de associação dizem respeito aos aspectos sociodemográficos que interferiram nas habilidades de leitura e numeramento, e no PCS e MCS de ribeirinhos usuários da APS de modalidade ribeirinha.

# Translation: Alfabetización funcional en salud y calidad de vida de pobladores ribereños en la atención primaria de salud*

**DOI:** 10.1590/1518-8345.7402.4439

**Authors:** Ana Kedma Correa Pinheiro, Rejane de Fátima Parada Viegas, Ingrid Bentes Lima, Ivaneide Leal Ataide Rodrigues, Sheila Nascimento Pereira de Farias, Laura Maria Vidal Nogueira

**Affiliations:** 1 Universidade Federal do Rio de Janeiro, Escola de Enfermagem Anna Nery, Rio de Janeiro, RJ, Brasil; 2 Becaria de la Fundação Amazônia Paraense de Amparo à Pesquisa (FAPESPA), Brasil; 3 Becaria de la Coordenação de Aperfeiçoamento de Pessoal de Nível Superior (CAPES), Brasil; 4 Universidade do Estado do Pará, Escola de Enfermagem Magalhães Barata, Belém, PA, Brasil; 5 Universidade do Estado do Pará, Departamento de Enfermagem Comunitária, Belém, PA, Brasil

**Keywords:** Alfabetización en Salud, Calidad de Vida, Factores Socioeconómicos, Salud Rural, Enfermería Rural, Atención Primaria de Salud

## Abstract

analizar la alfabetización funcional en salud y la calidad de vida relacionada con la salud de una población ribereña que utiliza la atención primaria, según variables sociodemográficas.

estudio analítico, transversal, con 312 usuarios de la estrategia salud de la familia ribereña. Los datos se recopilaron mediante la prueba de alfabetización en salud, *12-item Health Survey* y un cuestionario socioeconómico adaptado por los investigadores. Se realizaron pruebas de correlación de Spearman, Mann-Whitney, Kruskal-Wallis y regresión logística múltiple, se consideró p≤0,05.

el 65,7% presentó alfabetización funcional en salud inadecuada y el mayor riesgo lo tenían los hombres (p<0,001) con edades entre 40-49 (p=0,010) y 50-59 años (p=0,031), educación primaria incompleta (p<0,001) y completa (p=0,024), que vivían lejos del servicio de salud (p<0,001). La calidad de vida no mostró asociación con la alfabetización en salud, sin embargo, la menor calidad de vida presentó relación con el sexo femenino (p=0,049), educación primaria incompleta (p=0,016), uso de celulares con internet y radio (p= 0,013), edad avanzada (p<0,001), mayor número de hijos (p=0,002) y menor edad de inicio de las actividades laborales (p<0,001).

la alfabetización funcional en salud de los pobladores ribereños es inadecuada y no está asociada con la calidad de vida, sin embargo, ambas están influenciadas por el perfil sociodemográfico de los pobladores ribereños.

## Destacados

**(1)** La alfabetización funcional en salud de los pobladores ribereños es inadecuada. **(2)** La alfabetización en salud no se asoció con la calidad de vida. **(3)** La alfabetización en salud se asoció con aspectos sociodemográficos de los pobladores ribereños. **(4)** La calidad de vida se relacionó con el perfil sociodemográfico de los pobladores ribereños.

## Introducción

La Alfabetización Funcional en Salud (AFS) se refiere a la capacidad cognitiva de una persona para leer, interpretar y decodificar información disponible y transmitida en el servicio de salud, lo cual es importante para tomar decisiones terapéuticas y mantener el autocuidado y el bienestar^(1)^.

En el desarrollo de este estudio se adoptaron los conceptos del modelo teórico de Alfabetización en Salud (AS)^(2)^, que considera conocimientos, motivación y habilidades para mantener o mejorar la Calidad de Vida (CV). Se trata de un modelo que engloba tres grupos de factores, desde el más distal al proximal, que son: determinantes socioambientales, que involucran la demografía, la cultura y los sistemas sociales; factores situacionales que involucran apoyo familiar y medios de comunicación; y factores personales que incluyen variables socioeconómicas.

La calidad de vida abarca un concepto amplio y sus dimensiones incluyen los aspectos: físico; mental y social. Está directamente relacionada con la capacidad funcional, el bienestar socioeconómico y el grado de satisfacción de la persona. Los componentes físico y mental aportan variables importantes para evaluar la Calidad de Vida Relacionada con la Salud (CVRS)^(3)^.

Este es un tema que despierta interés global, como lo demuestra la evidencia científica obtenida a partir de la búsqueda realizada en siete bases de datos, que incluye el *Medical Literature Analysis and Retrieval System Online*, en la que se identificaron varios estudios que abordaron la AS y la CV, sin embargo, solo cinco se realizaron con poblaciones rurales y ninguno con poblaciones ribereñas.

En los estudios que asociaron AS y CV, se observaron repercusiones positivas relacionadas con la autopercepción del estado de salud^(4-9)^, la autogestión^(10-14)^, las intervenciones centradas en promover la salud^(15-16)^, la adherencia al tratamiento^(17)^, la salud física^(18-19)^ y salud mental^(20-22)^. Las repercusiones negativas estaban relacionadas con la falta de acceso a la información de salud, el empeoramiento de las condiciones clínicas y la mayor demanda de apoyo médico^(23-24)^.

Se destaca un estudio realizado en Alemania^(4)^, con personas atendidas en un sistema de salud integrado, similar al contexto de la Atención Primaria de Salud (APS), que confirmó que había asociación entre AS adecuada y CVRS alta, en zonas rurales. A pesar de las características geográficas de las zonas rurales, no se consideran ribereñas.

Otra iniciativa se refiere al estudio realizado en China^(20)^, con personas con prediabetes, en el contexto de la APS, que identificó una asociación entre AS adecuada y CVRS alta. Si bien se realizó con habitantes de zonas rurales, no es una comunidad ribereña.

En Brasil, en el estado de Pará, investigaciones sobre AFS revelaron que había asociación entre alfabetización inadecuada y bajo nivel educativo y bajos ingresos, lo que confirma que repercute en el déficit de autocuidado y la promoción de la salud de los usuarios de la APS^(1)^. Cabe destacar que Pará forma parte de la Amazonía brasileña, donde reside una importante población ribereña, cuyo contexto de vida es peculiar, lo que puede tener un impacto en los indicadores de morbilidad y mortalidad, y requiere una observación atenta del perfil sociodemográfico y del modo de vida^(25)^.

Los pobladores ribereños dependen casi exclusivamente del Sistema Único de Salud (SUS) y acuden al servicio más cercano a su domicilio, pero están condicionados por el entorno, dado que tienen dificultades para desplazarse porque los medios de transporte son precarios. El trabajo de los equipos de la APS en esas comunidades necesita garantizar resolución, sin embargo, enfrenta desafíos geográficos y un número reducido de profesionales con alta rotación debido a relaciones laborales precarias^(26)^.

Cabe señalar que las acciones de los equipos de la APS y gestores de salud deben basarse en la Política Nacional de Salud Integral de las Poblaciones del Campo, la Selva y del Agua (*Política Nacional de Saúde Integral das Populações do Campo, da Floresta e das Águas*)^(27)^ y en la Política Nacional de Atención Primaria (*Política Nacional de Atenção Básica,* PNAB)^(28)^ para satisfacer las necesidades específicas de los habitantes locales. En su rutina de trabajo, es desafiante e imprescindible que los profesionales de la salud utilicen vocabulario y material educativo integral apto para dichas poblaciones, lo que corresponde al área de la AS^(29)^.

Por lo tanto, el enfoque de este estudio es de gran interés para el avance de la ciencia, presenta evidencia científica de una población vulnerable con un modo de vida peculiar y se puede replicar en otros espacios geográficos que compartan características similares a las poblaciones ribereñas, especialmente en América Latina.

Para comprender mejor la relación entre AFS y CVRS en una comunidad ribereña de la Amazonia, este estudio tuvo como objetivo analizar la alfabetización funcional en salud y la calidad de vida relacionada con la salud en una población ribereña que utiliza la atención primaria, según variables sociodemográficas.

## Método

### Diseño del estudio

Estudio analítico y transversal, que se basa en el *Strengthening the Reporting of Observational Studies in Epidemiology* (STROBE)^(30)^ como guía para la redacción del manuscrito.

### Lugar y población del estudio

Se realizó en una zona ribereña del municipio de Abaetetuba, estado de Pará, Brasil, ubicado en el Baixo Tocantins, con una población de 158.188 habitantes, según el censo de 2022^(31)^. La región ribereña abarca 72 localidades, con aproximadamente 42 mil habitantes^(32)^.

El estudio se realizó en un territorio que forma parte del área de cobertura del equipo de Salud de la Familia Ribereña (eSFR), que atiende a usuarios de cuatro localidades, denominadas en este estudio de los ríos: X, Y, Z, W. La eSFR está ubicada en el río X y abarca ocho microáreas, dos de las cuales no cuentan con la cobertura de los Agentes Comunitarios de Salud (ACS) y pertenecen al río Z. Las localidades fueron elegidas porque la población recibe atención en la única eSFR de la Región.

Según el registro del Sistema de Territorialización del Sistema Único de Salud (e-SUS-territorio), el río X tiene 461 habitantes ≥ 18 años, el río Y, 133, el río Z, 118 y el río W, 668, en total son 1.380 usuarios, que reciben atención en la eSFR. Además, en el río Z aún hay residentes no incluidos en esos datos, porque no están registrados en el e-SUS-territorio, debido a que residen en áreas geográficas que no cuentan con la cobertura de los ACS.

### Criterios de selección

Se incluyeron usuarios mayores de 18 años, de ambos sexos, que supieran leer y escribir, residentes en la región ribereña del municipio de Abaetetuba, en territorio cubierto por la eSFR. Se excluyeron personas con limitaciones visuales y/o auditivas que les impidieran leer los instrumentos o incluso escuchar al entrevistador durante la recolección de datos, así como personas con discapacidades cognitivas o conductuales que dificultaran la participación en el estudio, previamente identificadas en los registros del e-SUS-territorio y/o autoinformadas por los participantes o familiares.

### Definición de la muestra

La muestra estuvo conformada por 312 usuarios, definidos mediante la técnica de muestreo Cochran^(33)^, considerando el tamaño de la población y un error de muestreo del 5%.

Se decidió incluir representantes de los cuatro ríos en el estudio, de forma proporcional, basándose en el registro de usuarios del e-SUS-territorio. También se consideraron los diferentes niveles educativos, según datos divulgados por el Instituto Brasileño de Geografía y Estadística (IBGE), que reconoce las siguientes proporciones: el 38,6% de la población corresponde a personas sin educación y con educación primaria incompleta; el 12,5% con educación primaria completa y secundaria incompleta; y el 48,8% educación secundaria completa o más^(34)^.

El muestreo fue no probabilístico por cuotas^(35)^, ya que la selección de los participantes se basó en la representación proporcional de las cuatro localidades, se consideró el número de personas residentes en el territorio correspondiente a los tres niveles educativos. Esta opción permitió obtener datos representativos de cada río.

Se incluyeron las cuatro ubicaciones para tener en cuenta la diversidad de la población, y la estratificación por nivel educativo, para evitar sesgos de muestreo que puedan afectar los niveles de alfabetización, dado que la educación se relaciona con la AS^(29,36)^.

### Instrumentos utilizados y variables de estudio

Se utilizó la Prueba de Alfabetización en Salud (PAS), validada en Brasil en 2019 y adaptada transculturalmente a la lengua portuguesa versión brasileña^(36-37)^, que consta de dos partes: evaluación del dominio de la aritmética con 17 ítems y evaluación de la lectura y comprensión con 50 artículos. Se eligió esa prueba porque permite evaluar los dos dominios. La consistencia interna fue de 0,953 y se aplicó según las recomendaciones de los autores^(36-37)^.

Los puntajes atribuidos a la PAS siguieron las recomendaciones del instrumento original^(37)^. Para calcularlos le asignó un punto a cada respuesta correcta y cero a las respuestas incorrectas y/o autodeclaración de no saber y/o no completarla. En cuanto a la parte numérica, la puntuación asignada, hasta 17 en el puntaje bruto, se multiplicó por 2,941, para convertirlo en una puntuación de cero a 50^(37)^.

No se ponderó la parte de lectura. Al final, los puntajes se calcularon mediante la suma de los dominios, y se clasificó como alfabetización adecuada los que alcanzaron entre 75 y 100 puntos, alfabetización limitada los que obtuvieron entre 60 y 74 y alfabetización inadecuada los que se mantuvieron en el rango de cero a 59 puntos^(36)^.

Para medir la CVRS se utilizó el *12-itemsHealth Survey* (SF-12), versión 1.0, creada en 1994^(38)^ y validado en Brasil en 2004^(39)^, compuesto por 12 ítems y dividido en dos componentes, el físico, llamado *Physical Component Summary*(PCS), y el mental, definido como *Mental Component Summary* (MCS). Cada componente tiene cuatro dominios: el PCS incluye salud general, dolor, capacidad funcional y aspecto físico; y, el MCS incluye los dominios aspecto emocional, salud mental, vitalidad, además del aspecto social^(39)^.

Se eligió el SF-12 porque es un instrumento que se usa para evaluar la salud general, con un formato simple, breve, de rápida aplicación, ampliamente utilizado para medir de manera precisa y eficiente la salud física y mental en investigaciones con la población general. Presenta un alto grado de confiabilidad, tiene un coeficiente alfa de Cronbach igual a 0,836^(40-41)^.

El cálculo de los puntajes del SF-12 siguió las recomendaciones del instrumento original^(38)^, que presenta una escala graduada tipo Likert, cuya puntuación varía según la composición de la pregunta, con opción de dos a seis respuestas, que van de cero a 100, y los puntajes más altos se asocian con mejores niveles de calidad de vida. El análisis de cada componente se realizó por separado, no se asignó un puntaje total^(38-39)^, sino que se agruparon en PCS y MCS. Para definir el puntaje de los componentes, se consideró el algoritmo de cálculo de la puntuación, considerando la media de la población norteamericana en 1998, que ha sido adoptada en estudios brasileños e internacionales^(38-41)^.

Para determinar el perfil sociodemográfico se utilizó un cuestionario elaborado por el centro de investigación y la confederación nacional de trabajadores y trabajadoras agrícolas, estructurado para realizar el proyecto “*Escuta itinerante: acesso das populações do campo e da floresta ao SUS*”^(42)^. El instrumento tiene dos partes: la primera se refiere al perfil y modo de vida; producción y trabajo en el campo; y, sistema de salud. La segunda parte corresponde a la identidad de género y edad de los participantes.

De este instrumento se extrajeron siete preguntas que se relacionan con el presente estudio y permitieron obtener datos sobre el perfil sociodemográfico de los pobladores ribereños. Estas preguntas se tratan en la primera parte del cuestionario, seis de las cuales se refieren al perfil/forma de vida, que incluye género, edad, ubicación, educación, número de hijos y medios de comunicación. La otra pregunta se refiere a la producción/trabajo en el campo, lo que permitió investigar la edad de inicio de las actividades laborales. Estas variables fueron adaptadas según las investigaciones realizadas con la población ribereña de la Amazonía^(3,43)^.

Se realizó un estudio piloto con pobladores ribereños usuarios de la APS, residentes en otras localidades del municipio, lo que permitió adaptar los términos de la PAS a la cultura local, considerando el significado semántico. También fue importante para orientar la capacitación de los entrevistadores y planificar el trabajo de campo.

Los ajustes realizados a la PAS se refieren a la inserción de algunos términos explicativos de palabras técnicas, con el objetivo de facilitar la comprensión de los participantes. En la prueba de aritmética se insertaron palabras para explicar los siguientes términos: cápsula = pastilla; antipirético = medicamento para tratar la fiebre; y tylenol = paracetamol. En la prueba de comprensión lectora se agregaron las siguientes explicaciones: persona que tiene asma = asmático; medicamento para la acidez estomacal = antiácido; enfermedad ósea = osteoporosis; bajo nivel de azúcar en sangre = hipoglucemia; y enfermedad mental = esquizofrenia.

### Recolección de datos

La recolección de datos se realizó entre febrero y mayo de 2021, en el edificio de la eSFR, mientras esperaban para ser atendidos y durante las visitas domiciliarias programadas por los ACS, a criterio de los participantes. La duración media de las entrevistas fue de veintitrés minutos.

La PAS fue autocompletada en la parte de comprensión y lectura, en la que el entrevistado llenó los vacíos seleccionando la opción más adecuada para el texto, evaluada por los fragmentos: A que contenía instrucciones para el procedimiento de radiografía, el B que incluía el texto inicial de la legislación brasileña sobre el SUS y el C que consistía en un formulario de consentimiento para la realización de procedimientos. La parte de aritmética contó con la ayuda del entrevistador, quien les dio tarjetas con información numérica y luego realizó las preguntas previstas en el instrumento. Se registró el tiempo de aplicación de cada parte de la PAS^(36)^. El SF-12 fue administrado con ayuda del entrevistador.

Los entrevistadores fueron la investigadora principal y cinco estudiantes de la carrera de grado en Enfermería de una universidad pública estadual, que cursaban la asignatura Enfermería y Poblaciones Tradicionales de la Amazonia, previamente capacitados y supervisados por la investigadora principal.

Los obstáculos experimentados durante la recolección de datos involucraron limitaciones logísticas, tales como: navegación en pequeños ríos sedimentados y en presencia de oleaje, paso a través de pozos, dificultad para conseguir transporte seguro para el equipo y horarios de recolección de datos que entraban en conflicto con el turno de trabajo de los pobladores ribereños.

Para superar los obstáculos, el equipo de investigación contó con el apoyo de los líderes de las comunidades que hospedaron a los investigadores en sus casas, lo que les permitió permanecer en el territorio y aprovechar mejor el tiempo de recolección de datos. La colaboración también permitió contratar una embarcación de mediano porte que permitía sortear el oleaje.

### Procesamiento y análisis de datos

Los datos se ingresaron con doble entrada en el *Statistical Package for the Social Sciences*, versión 21. Se utilizó la correlación de Spearman para comparar puntuaciones, puntajes y edades. Para evaluar la normalidad se utilizó la prueba de Shapiro-Wilk. En el análisis bivariado de esos puntajes con las variables sociodemográficas se aplicaron las pruebas de Mann-Whitney y Kruskal-Wallis, que permitieron comparar los puntajes con las variables categóricas.

Se aplicó el modelo de regresión logística para evaluar la asociación entre las variables de interés (género, edad, localidad, educación, número de hijos, medios de comunicación e inicio de trabajo) y el resultado AFS inadecuada. Las variables que presentaron asociación significativa en el análisis de regresión logística bivariada permanecieron en el modelo ajustado. Todos los análisis se realizaron en el paquete estadístico R, versión 4.1.1, considerando un nivel de significación de 0,05.

### Aspectos éticos

El estudio siguió las Resoluciones 466/12 y 580/18 del Consejo Nacional de Salud/Ministerio de Salud brasileño, y fue aprobado por el Comité de Ética en Investigación de la Carrera de Grado en Enfermería de la Universidad Estadual de Pará, bajo dictamen 4.517.829 del 1 de febrero de 2021.

## Resultados

De los 312 pobladores ribereños que integraron la muestra, el 67,0% (n=209) eran del sexo femenino y la mayoría tenía de dos a tres hijos, con un promedio de 2,18±1,999, y oscilaba entre cero y 13 hijos. La edad promedio alcanzó 35,03±13,397 años, y oscilaba entre 18 y 78 años, la mayor frecuencia (71,5%) era entre 18 y 39 años. El 61,5% de la muestra utilizaba teléfono celular (Tabla 1).

La media de inicio de la vida laboral fue de 12,51±4,518 años y osciló entre 3 y 31 años, la mitad de la muestra inició las actividades laborales entre los 3 y 13 años (n=156; 50,0%). La media del puntaje total de la PAS fue 52,235±21,638, con una variación de 5,941-99,997. Del total, 205 (65,7%) tenían AFS inadecuada, 54 (17,3%) limitada y 53 (17,0%) adecuada. La puntuación media de la AFS total (p<0,001) presentó una reducción proporcional al compromiso de la alfabetización. Se identificaron mayores promedios de AFS en el sexo femenino (p<0,001), lugar de residencia en el Río X (p<0,001) y uso de celular con internet (p<0,001). Se identificaron promedios más bajos en los residentes del río W (p<0,001) sin acceso a dispositivos de comunicación (p<0,001). La AFS se redujo con el aumento del número de hijos (p<0,001) y la edad (p<0,001), y aumentó con el aumento del nivel educativo (p<0,001) y de la edad de inicio de las actividades laborales (p<0,001) (Tabla 1).

**Tabla 1 -:** Análisis de la alfabetización funcional en salud según variables sociodemográficas de los pobladores ribereños (n* = 312). Abaetetuba, PA, Brasil, 2021

			**Puntaje de la PAS** ^†^		
**Variable**	**Categoría**	n* (%) ^‡^	**Media**	**Error estándar**	**valor p** ^§^
**Sexo**					
	Femenino	209 (67,0%)	55,530	1,531	<0,001
	Masculino	103 (33,0%)	45,551	1,873	
**Número de hijos**					
	0	61 (19,6%)	59,157	2,723	<0,001
	1	66 (21,2%)	56,756	2,353	
	2 a 3	123 (39,4%)	51,940	1,997	
	4 a 13	58 (18,6%)	40,563	2,540	
	NI ^||^	4 (1,3%)	50,410	10,754	
**Edad (años)**					
	18 a 29	122 (39,1%)	59,462	1,816	<0,001
	30 a 39	101 (32,4%)	53,331	2,178	
	40 a 49	37 (11,9%)	44,906	3,079	
	50 a 59	24 (7,7%)	38,150	4,291	
	60 a 78	24 (7,7%)	35,775	3,078	
	NI ^||^	4 (1,3%)	55,190	10,176	
**Localidad**					
	Río X	111 (35,6%)	59,276	2,118	<0,001
	Río Y	33 (10,6%)	49,232	3,282	
	Río Z	29 (9,3%)	53,783	3,725	
	Río W	139 (44,6%)	47,003	1,747	
**Educación**					
	EPI ^¶^	132 (42,3%)	37,432	1,383	<0,001
	EPC**	32 (10,3%)	53,138	3,135	
	ESI ^††^	30 (9,6%)	56,120	3,164	
	ESC ^‡‡^	85 (27,2%)	64,998	1,854	
	ESI ^§§^	15 (4,8%)	78,978	4,232	
	ESC ^||||^	18 (5,8%)	70,158	4,443	
**Comunicación**					
	Teléfono celular	192 (61,5%)	46,839	1,434	<0,001
	Celular e internet	110 (35,3%)	62,982	1,995	
	Celular, internet y radio	3 (1,0%)	44,743	9,127	
	No tiene	3 (1,0%)	39,508	7,172	
	NI ^||^	4 (1,3%)	30,881	8,662	
**Inicio del trabajo**					
	3 a 13 años	156 (50,0%)	45,240	1,544	<0,001
	14 a 17 años	60 (19,2%)	52,62	2,629	
	18 a 31 años	39 (12,5%)	67,310	3,038	
	Nunca trabajó	55 (17,6%)	61,030	3,081	
	NI ^||^	2 (0,6%)	50,440	20,794	

*n = Número de participantes; ^†^PAS = Prueba de Alfabetización en Salud; ^‡^% = Frecuencia porcentual; ^§^valor p = Prueba de Mann-Whitman para variables con dos categorías y de Kruskal-Wallis para las demás, significativa para p≤0,05; ^||^NI = No Informado; ^¶^EPI = Educación Primaria Incompleta; **EPC = Educación Primaria Completa; ^††^ESI = Educación Secundaria Incompleta; ^‡‡^ESC = Educación Secundaria Completa; ^§§^ESI = Educación Superior Incompleta; ^||||^ESC = Educación Superior Completa

En cuanto a la CVRS, el PCS y el MCS presentaron una media de 43,461±10,176 y 47,752±9,633, una mediana de 44,730 y 50,191, una variación de 14,063-62,075 y 19,317-66,982, respectivamente. El PCS (p=0,090) y el MCS (p=0,776) no mostraron asociación con la AFS, sin embargo, el sexo femenino (p=0,049) tuvo diferencia estadística con menor CVRS en el MCS (Tabla 2).

En relación al PCS, se identificó menor CVRS en los participantes con educación primaria incompleta (p=0,016), que utilizaban celulares con internet y radio (p=0,013). Sin embargo, aquellos que no utilizaban dispositivo de comunicación tuvieron mejor CV en el PCS (p=0,013). El PCS de la CVRS se redujo con el aumento del número de hijos (p=0,002), el aumento de la edad hasta los 59 años (p<0,001) y disminución de la edad de inicio de las actividades laborales (p<0,001) (Tabla 2).

**Tabla 2 -:** Análisis de la calidad de vida relacionada con la salud según la alfabetización funcional en salud y las características sociodemográficas de los pobladores ribereños (n* = 312). Abaetetuba, PA, Brasil, 2021

		**PCS** ^†^			**MCS** ^‡^		
**Variable**	**Categoría**	**Media**	**Error estándar**	**valor p** ^§^	**Media**	**Error estándar**	**valor p** ^§^
**AFS** ^||^							
	Inadecuado	42,495	0,749	0,090	47,744	0,667	0,776
	Limitado	45,724	1,246		48,239	1,424	
	Adecuado	44,892	1,161		47,285	1,267	
**Sexo**							
	Femenino	43,346	0,737	0,813	47,042	0,655	0,049
	Masculino	43,695	0,903		49,192	0,970	
**Número de hijos**							
	0	46,212	1,044	0,002	46,674	1,216	0,084
	1	46,235	1,161		49,644	1,237	
	2 a 3	42,218	0,949		48,063	0,859	
	4 a 13	40,145	1,446		46,603	1,218	
	No informado	42,046	4,062		40,040	4,766	
**Edad (años)**							
	18 a 29	46,197	0,835	<0,001	48,121	0,887	0,180
	30 a 39	44,823	0,919		47,779	0,917	
	40 a 49	39,204	1,767		46,036	1,562	
	50 a 59	34,553	2,197		50,625	1,844	
	60 a 78	39,388	1,935		44,526	2,275	
	No informado	42,899	5,886		53,780	2,088	
**Educación**							
	EPI ^¶^	41,241	0,901	0,016	47,605	0,845	0,364
	EPC**	43,790	2,066		45,994	1,620	
	ESI ^††^	46,978	1,657		48,250	1,488	
	ESC ^‡‡^	44,883	0,998		48,710	1,099	
	ESI ^§§^	46,860	2,287		44,267	2,590	
	ESC ^||||^	43,750	2,526		49,504	2,240	
**Comunicación**							
	Teléfono celular	42,006	0,763	0,013	47,991	0,697	0,393
	Celular e internet	45,876	0,853		47,874	0,904	
	Celular, internet y radio	37,264	5,894		38,000	7,060	
	No tiene	54,009	3,015		43,177	5,167	
	No informado	43,647	5,366		43,650	5,578	
**Inicio del trabajo**							
	3 a 13 años	40,964	0,877	<0,001	47,468	0,797	0,816
	14 a 17 años	44,943	1,178		47,189	1,317	
	18 a 31 años	45,213	1,473		49,666	1,389	
	Nunca trabajó	47,850	1,032		47,881	1,157	
	No informado	38,956	1,476		45,914	13,167	

*n = Número de participantes; ^†^PCS = *Physical Component Summary;* ^‡^MCS = *Mental Component Summary*; ^§^valor p = Prueba de Mann-Whitman para variables con dos categorías y de Kruskal-Wallis para las demás, significativa para p≤0,05; ^||^AFS = Alfabetización Funcional en Salud; ^¶^EPI = Educación Primaria Incompleta; **EPC = Educación Primaria Completa; ^††^ESI = Educación Secundaria Incompleta; ^‡‡^ESC = Educación Secundaria Completa; ^§§^ESI = Educación Superior Incompleta; ^||||^ESC = Educación Superior Completa

La Tabla 3 indica que cuanto mejor es el PCS, mayor es la habilidad de lectura (p≤0,01), mayor es el puntaje en el fragmento A (p≤0,05) y B (p≤0,05) de la lectura. Mientras que, cuanto peor es el PCS, mayor es la edad (p≤0,01) y más tiempo le dedica a completar el fragmento A (p≤0,05) y todos los fragmentos de lectura (p≤0,05). No hubo correlación significativa entre los MCS y los ítems de medición de la PAS.

**Tabla 3 -:** Correlación de Spearman entre los componentes físicos y mentales de la calidad de vida relacionada con la salud y edad y la prueba de alfabetización en salud de pobladores ribereños (n* = 312). Abaetetuba, PA, Brasil, 2021

**Variables**	**PCS** ^†^	**MCS** ^‡^
Edad	-0,334 ^§^	-0,059
Tiempo fragmento A de la lectura	-0,172 ^||^	-0,019
Tiempo fragmento B de la lectura	0,053	0,021
Tiempo fragmento C de la lectura	0,09	0,011
Tiempo total de lectura	-0,152 ^||^	-0,025
Puntaje del fragmento A de la lectura	0,159 ^||^	0,055
Puntaje del fragmento B de la lectura	0,150 ^||^	0,065
Puntaje del fragmento C de la lectura	0,142	-0,001
Puntaje total de lectura	0,189 ^§^	0,073
Puntaje de aritmética	0,06	0,082
Tiempo de la prueba de aritmética	-0,115	0,017
Puntaje total de la Prueba de alfabetización en salud	0,140	0,084

*n = Número de participantes; ^†^PCS = *Physical Component Summary*; ^‡^MCS = *Mental Component Summary*; ^§^≤0,01; ^||^>0,01 - ≤0,05

Los modelos bivariado y ajustado se presentan en la Tabla 4. El ajuste del modelo indica que el uso de un teléfono celular con internet puede ser un factor protector para la AFS inadecuada. Además, las personas del sexo masculino, con edades entre 40 y 49 y 50 y 59 años, más alejados del servicio de salud (Rio W), con educación primaria (completa e incompleta) presentaron mayor riesgo de tener AFS inadecuada.

**Tabla 4 -:** Asociación entre alfabetización inadecuada y características sociodemográficas (n* = 312). Abaetetuba, PA, Brasil, 2021

	**Modelo bivariado**		**Modelo ajustado**	
**Característica** s	**OR** ^†^ **(I** C **95%)** ^‡^	**valor p** ^§^	**OR** ^†^ **(I** C **95%)** ^‡^	**valor p** ^§^
Sexo (referencia = Femenino)				
Masculino	2,96 (1,72; 5,29)	<0,001	4,42 (2,11; 9,73)	<0,001
Número de hijos (referencia=0 hijos)				
1 hijo	1,32 (0,66; 2,66)	0,438		
2 o 3 hijos	2,5 (1,33; 4,74)	0,005		
4 hijos o más	4,79 (2,16; 11,3)	<0,001		
Edad (referencia=18 a 29 años)				
30 a 39 años	1,75 (1,02; 3,01)	0,043	1,51 (0,72; 3,20)	0,278
40 a 49 años	7,98 (2,95; 28,0)	<0,001	6,01 (1,68; 26,4)	0,010
50 a 59 años	6,77 (2,19; 29,7)	0,003	5,92 (1,29; 34,6)	0,031
60 años o más	10,6 (2,96; 68,3)	0,002	3,66 (0,67; 31,9)	0,174
No informado	0,97 (0,11; 8,28)	0,974	0,94 (0,05; 14,3)	0,963
Localidad (referencia=Rio X)				
Río Y	2,18 (0,97; 5,19)	0,066	1,81 (0,61; 5,62)	0,290
Río Z	1,55 (0,68; 3,67)	0,305	1,6 (0,45; 5,74)	0,468
Río W	3,17 (1,85; 5,50)	<0,001	3,7 (1,78; 7,94)	<0,001
Educación (referencia=Superior completa)				
Superior incompleta	0,31 (0,04; 1,64)	0,195	0,43 (0,05; 2,91)	0,410
Primaria completa	5,11 (1,53; 18,9)	0,010	5,61 (1,31; 26,8)	0,024
Primaria Incompleta	31 (9,67; 112)	<0,001	16,5 (4,35; 70,3)	<0,001
Secundaria completa	1,21 (0,42; 3,76)	0,731	1,03 (0,29; 3,99)	0,959
Secundaria Incompleta	3 (0,91; 10,8)	0,078	2,91 (0,69; 13,4)	0,156
Medios de comunicación (referencia=Teléfono celular)				
Teléfono celular e internet	0,25 (0,15; 0,40)	<0,001	0,47 (0,23; 0,94)	0,033
Teléfono celular, internet y radio	0,61 (0,06; 13,4)	0,692	2,47 (0,09; 149)	0,645
No tiene	4791233 (0,00; Inf ^||^ )	0,986	3900155 (0,00; Inf ^||^ )	0,985
Inicio del Trabajo (referencia=3 a 13 años)				
14 a 17 años	0,6 (0,31; 1,20)	0,141		
18 a 31 años	0,13 (0,06; 0,27)	<0,001		
Nunca trabajó	0,22 (0,11; 0,41)	<0,001		

*n = Número de participantes; ^†^OR = *Odds Ratio;* ^‡^IC = Intervalo de confianza (95%); ^§^valor p = Prueba de Mann-Whitman para variables con dos categorías y de Kruskal-Wallis para las demás, significativa para p≤0,05; ^||^Inf = Infinito

## Discusión

La AFS resultó ser inadecuada en la mayoría de los participantes. Un estudio realizado en Brasil^(36)^, que validó la PAS, identificó que aproximadamente la mitad de los 302 participantes tenían AFS inadecuada o limitada, debido a cuestiones sociodemográficas, especialmente por el aumento de la edad y la reducción de los años de estudio.

Por el contrario, una investigación realizada en zonas rurales de Alemania concluyó que el 61,9% tenía suficiente AS. Si bien la muestra estuvo conformada por grupos poblacionales con perfil social y educativo desfavorable, el nivel de AS se debía a la inversión en prevención, promoción de la salud y empoderamiento de la gestión, que tuvo efectos positivo en las personas^(4)^.

Según estas discusiones y el perfil sociodemográfico de los pobladores ribereños, se justifica la prevalencia de habilidades básicas de aritmética y comprensión lectora inadecuadas, necesarias para comprender la información de salud, dado que la distancia y las limitaciones logísticas para acceder a los servicios de salud pueden interferir con el deterioro de la AFS. Además, las vulnerabilidades sociales presentes en el contexto local pueden afectar el uso de la información proporcionada por el servicio de salud y repercutir en las decisiones individuales.

Por otro lado, la literatura nacional e internacional sobre AS también publicó que había una mayor proporción de mujeres, ello indica que predomina el sexo femenino^(1,15,36,44-45)^, lo que puede atribuirse a que las mujeres acuden más a los servicios de salud y a que los hombres se encuentran ausentes en los ambientes familiares cuando se realiza la recolección de datos debido a las actividades laborales.

Se considera que la mujer ribereña amazónica es un eslabón importante de la red de apoyo y cuidado familiar, acumula actividades domésticas sin compensación de ingresos, tiene un perfil de bajo nivel educativo, embarazo adolescente, altas tasas de gestación y fecundidad. Estos resultados pueden orientar la creación de proyectos terapéuticos importantes en el contexto de la AS de mujeres ribereñas de la Amazonia^(43)^.

En lo que respecta a la AFS, las mujeres tenían mejores niveles de habilidades aritméticas y de comprensión lectora y menor riesgo de tener AFS inadecuada, resultados que coinciden con los de un estudio realizado en Japón^(45)^, en el que las mujeres tenían mejores habilidades de AS para la toma de decisiones. Aunque el estudio fue realizado en otro país, hay similitudes con el contexto ribereño de la Amazonía de Pará, en el que las mujeres asumen el rol de principales cuidadoras en la familia, y son más propensas a aplicar medidas de promoción para la salud, además del rol de gestión de las consultas y los medicamentos y mayor presencia en los servicios de salud.

Por otro lado, las mujeres presentaron mayor deterioro de la CV mental, en la que destaca la dimensión del universo femenino que involucra actividades propias del modelo patriarcal, según el cual las mujeres son responsables del cuidado de los hijos y del hogar, situaciones que afectan la estabilidad emocional y psicológica, que, incluso, pueden desencadenar cuadros de depresión^(46)^.

En cuanto a la fecundidad, se identificó que el número promedio de hijos equivale al valor de reemplazo poblacional de 2,1 hijos por mujer, superando las cifras registradas en Brasil, que corresponden a 1,4 hijos por mujer, según una investigación de las Naciones Unidas, que admitió que las personas con menor nivel educativo, más pobres, de zonas rurales y de la región norte de Brasil tienen mayor tasa de fecundidad^(47)^.

Se encontró una relación inversa entre la AFS y el número de hijos, ya que cuanto menor era el número de hijos, mejor era la AFS y quienes no tuvieron hijos alcanzaron una AFS aún mayor. Con respecto a eso, una investigación de salud realizada en comunidades ribereñas del Amazonas^(43)^ detectó que las mujeres tenían sus primeros embarazos durante la adolescencia y alta fecundidad, por lo que se debería considerar adoptar medidas de educación reproductiva valiéndose de la contribución teórica de la AS y así lograr mejor calidad de vida.

La evaluación de la CV física permitió identificar una asociación con el número de hijos debido a que se les exige realizar al mismo tiempo las actividades de cuidado, las laborales y las tareas domésticas que requieren esfuerzo físico. En este sentido, un estudio de revisión sistemática señaló que el número de hijos influye negativamente en la CV de los cuidadores familiares en lo que respecta al aspecto emocional y la vitalidad^(48)^.

En cuanto a las particularidades de la edad, se comprobó que la frecuencia de participantes se redujo con el aumento de la edad, que hubo una mayor proporción de participantes adultos jóvenes, que se acercaba a la edad promedio obtenida en un estudio de AS y CV, realizado en zona rurales y urbanas de China^(49)^. Además, se observó que había una relación inversa entre edad y AS, es decir, a mayor edad, peor AS y mayor riesgo de tener AFS inadecuada, lo que coincide con investigaciones realizadas en Brasil sobre la influencia de la AS en pacientes con enfermedad coronaria^(50)^.

Este escenario puede deberse a limitaciones inherentes al proceso de envejecimiento, que pueden comprometer la capacidad de asimilar información, influenciada por variables como los años de estudio y los hábitos de lectura. A medida que aumenta la edad, la capacidad cognitiva se ve afectada, y disminuye la capacidad para comprender conocimientos sobre salud^(45,51)^, debido a que la limitación para entender los cuadros de enfermedad y las formas de trabajo que tienen los pobladores ribereños con: baja educación, exposición a riesgos ergonómicos derivados de las actividades que requieren gran esfuerzo físico, posturas inadecuadas, jornadas laborales prolongadas, monotonía, repetitividad e imposición de una rutina intensa.

La falta de escuelas en algunas localidades y la restricción del acceso a la educación superior pública pueden potenciar el limitado conocimiento sobre salud, considerada el remanente histórico de una educación precaria y menos accesible, especialmente para personas vulnerables^(52)^.

Por lo tanto, la educación está directamente relacionada con la AS, es decir, cuanto más alta es, mejor es la AS. Cuanto menor nivel educativo tiene la persona, mayor riesgo tiene de presentar AFS inadecuada, y eso se relacionada con el nivel de desarrollo del país, dado que a mayor desarrollo, mejores son las tasas de escolarización y AS^(53)^.

En este estudio, las personas que vivían más lejos del servicio de APS tenían peor AFS y mayor riesgo de presentar AFS inadecuada que los que vivían en la localidad en la que se implementa el servicio, debido al difícil acceso al servicio y, por ende, acudían menos para recibir atención. Por consiguiente, la asistencia a los servicios de salud afecta el nivel de AFS de dichas poblaciones^(54)^ y el acceso limitado a la información se ha asociado con condiciones y contextos de salud desfavorables^(55)^.

La localidad más distante, aunque cuente con la cobertura de ACS, es una región conocida por la presencia de oleaje y difícil traslado debido al intenso tráfico de embarcaciones en la ruta hacia la eSFR. Las poblaciones ribereñas tienen limitaciones para acceder al servicio de salud debido a las particularidades de accesibilidad y a los desafíos geográficos, que se caracterizan por la presencia de oleaje, la falta de transporte fluvial y la dinámica de las corrientes^(32)^. Esta realidad contribuye a que estas personas estén excluidas de las acciones y servicios de salud e indica que es necesario que la APS lleve a cabo acciones para mejorar el nivel de AS y de CV^(26)^.

Es necesario evaluar el proceso salud-enfermedad de los pobladores ribereños en el contexto de la diversidad cultural y de las formas de vida^(29)^. Por lo tanto, es fundamental que la APS aproveche las particularidades y la cultura local para promover la AS de las personas, utilizando información relevante y que los pobladores consideren importantes para su realidad, que favorezca el bienestar y la adherencia a los tratamientos y, por ende, promueva la CV.

Al observar las peculiaridades de la vida ribereña, se constató que el medio de comunicación más utilizado es el teléfono celular con o sin internet, al igual que en una investigación del IBGE, que en 2019 confirmó en el 94,0% de los hogares del país había teléfono celular. Cabe aclarar que hay restricciones en el uso de internet en zonas rurales, principalmente debido a la dificultad para adquirir un dispositivo compatible por el alto costo y la falta de disponibilidad de señal de internet en la zona del hogar^(56)^.

Además, en muchas comunidades ribereñas el acceso a la información es limitado porque no disponen de electricidad estable^(25)^. El acceso a medios de comunicación con mayor desarrollo tecnológico favoreció la AFS de los pobladores ribereños, y se considera un factor protector de la AFS inadecuada, ya que quienes utilizaban un celular con internet tenían mejor AFS que los que no tenían dispositivo o solo usaban el teléfono celular.

Debido al desarrollo acelerado de la tecnología de comunicación electrónica y la gama de información vinculada a ella, es necesario mejorar la AS digital o *eHealth literacy* y así darles herramientas a los pobladores ribereños para que accedan y utilicen información de salud relevante a través de medios electrónicos^(15)^.

La calidad de vida física de quienes usaban radio y teléfonos celulares con internet se vio más comprometida y la de quienes no tenían un dispositivo de comunicación estuvo menos afectada. Ello indica que el avance de las tecnologías de la información en las regiones rurales, junto con el uso excesivo, genera dependencia tecnológica y puede producir inactividad física. Esta situación puede verse intensificada por los reducidos ambientes de interacción y espacios de ocio en el territorio, además del distanciamiento físico producido por la pandemia de COVID-19, factores que afectan la salud física.

Es un hecho que la transformación producida por internet se refleja en una reducción de la convivencia en la comunidad, sin embargo, permite incorporar tecnologías de atención, como la telesalud y las teleconsultas. Las mismas acortan las distancias geográficas, y contribuyen a la efectividad y resolución de la atención, especialmente en áreas con difícil acceso a servicios de salud, como las comunidades ribereñas en la Amazonía, lo que puede mejorar los niveles de AS y tener un impacto positivo en la CV física^(57)^.

Además, en el contexto de la vida ribereña, la inserción laboral temprana de niños y adolescentes se destaca como resultado de la negación y naturalización de la explotación del trabajo infantil bajo el argumento de que aumenta los ingresos familiares y les da la oportunidad de aprender con la práctica^(58)^. Sin embargo, las medidas gubernamentales se imparten de manera contradictoria sobre los niveles de trabajo infantil en los estados brasileños, las inspecciones no son suficientes y la política de transferencia de ingresos presenta limitaciones para combatir el trabajo infantil^(59)^.

El trabajo infantil se relacionó con una menor CV en el PCS, debido a la exposición a actividades de gran esfuerzo físico, como: agricultura de subsistencia y pesca artesanal. Dicha exposición puede tener consecuencias para la salud física, ya que implica trastornos del sistema musculoesquelético y accidentes laborales, que afectan la calidad de vida^(60)^.

Al analizar la edad de inicio de las actividades laborales, se comprobó que había una relación directa con la AFS, y se destaca que los niños y adolescentes que ingresaron tempranamente al mundo laboral presentaron peor AS que los que comenzaron en la fase de aprendiz o a partir de los 18 años. Esto se debe al deterioro intelectual que se refleja en escaso conocimiento, poca motivación y reducidas habilidades aritméticas y de lectura, y lleva a la deserción escolar y compromete la toma de decisiones de salud^(61)^.

La AS se identifica como un área de acción clave para alcanzar la agenda de desarrollo sostenible 2030, incluida en el octavo objetivo de desarrollo sostenible que indica que es necesario que haya mejores condiciones laborales y crecimiento económico. Por eso, mejores niveles de AS les permiten a los trabajadores rurales exigir oportunidades y mejores condiciones laborales^(61)^.

En cuanto a las oportunidades de trabajo e ingresos, están vinculadas al agua y la selva, que constituyen un medio de subsistencia. Viven de la agricultura familiar y la pesca artesanal que, sumadas a su cultura, configuran su subjetividad. A pesar de que la lógica capitalista considera que la pesca y la agricultura son insuficientes e insostenibles producen soberanía alimentaria y abastecen el comercio regional^(25)^.

En esta investigación, no se asociaron significativamente los componentes de la CVRS con diferentes niveles de AFS, como ocurrió en otros estudios que abordaron la AS y la CV, a saber: un estudio que midió el impacto de la AS en pacientes sometidos a terapia de reemplazo renal, asociado con los aspectos cognitivos, la adherencia a la medicación y la CV^(62)^; un estudio realizado con personas vulnerables que analizó la AS en el control de la diabetes en coreano-americanos^(10)^; un estudio que evaluó la relación entre AS y el miedo de las personas al *Coronavirus Disease* 2019 (COVID-19)^(63)^; y un estudio que analizó la relación entre AS, estilos de vida que promueven la salud y calidad de vida en estudiantes universitarios^(64)^.

Por otro lado, iniciativas internacionales han identificado esta relación, según un estudio que midió las interrelaciones entre demografía, AS, estado de salud autopercibido y CV de inmigrantes coreanos y vietnamitas^(5)^; un estudio que correlacionó AS con CV y estado de salud en comunidades rurales de Alemania^(4)^ y una investigación que analizó la CVRS de adolescentes y sus padres con AS en el contexto del COVID-19^(65)^.

El retrato sociodemográfico de los pobladores ribereños revela que es necesario mapear la AS para optimizar el conocimiento y la comunicación en salud, además de identificar dificultades para comprender la información recibida en los servicios de la APS. Por lo tanto, es importante que el servicio reconozca que la AS es un determinante social de la salud necesario para comprender las condiciones sociodemográficas y el modo de vida de las personas.

Se admite que la investigación sobre AFS con personas vulnerables aún está mal descrita en la literatura nacional e internacional, lo que limita comparar los resultados. Otra limitación se relaciona con el instrumento de recolección de datos que incluye textos autocompletados y ajenos a la realidad ribereña, lo que puede haber provocado sesgo de llenado.

Sin embargo, el aporte de este estudio radica en que contribuye a la planificación locorregional de la salud y de acciones educativas en la APS, especialmente para los enfermeros, orientadas a la implementación de prácticas compatibles con los niveles de AFS de los usuarios, que permitan mejorar la CV.

La investigación podría contribuir a promover intervenciones en el servicio, así como incentivar que se incluya el abordaje de la AS en los currículos de las carreras de grado y posgrado en salud. También puede favorecer un potencial avance en la Política Nacional de Salud Integral de las Poblaciones del Campo, la Selva y del Agua, al incorporar la perspectiva de la intersectorialidad para promover la AS y la CV, articulando el conocimiento tradicional, políticas de salud pública y educación. De esa forma, se vislumbra la implementación equitativa y resolutiva de acciones de educación para la salud, a través de estrategias adaptadas al modo de vida de las personas.

## Conclusión

Este estudio pionero en Brasil, que evaluó la AFS y la CVRS de pobladores ribereños usuarios de la modalidad ribereña de APS, demostró que no hay asociación entre la AFS y PCS y MCS de la CVRS, lo que permitió identificar que la AFS y la CVRS se relacionan con aspectos sociodemográficos.

Los pobladores ribereños de la APS presentaron AFS inadecuadas, lo que indica que es necesario efectivizar/implementar políticas públicas y planificación estratégica específica para brindar información de salud significativa y adecuada al perfil sociodemográfico de dichas poblaciones, con el fin de generar un cambio en la autogestión en salud.

El retrato sociodemográfico de los pobladores ribereños revela que los hombres, adultos mayores, que viven más alejados del servicio, sin acceso a internet, con menor nivel educativo y que sufrieron explotación mediante el trabajo infantil, tuvieron peor AFS y requieren una comunicación de salud simple, objetiva y significativa por parte de la APS, acorde a su forma de vida.

Para que las intervenciones mejoren la CV de los pobladores ribereños, es necesario considerar que su perfil presenta una CV física con mayor deterioro en adultos mayores, con baja escolaridad, acceso a medios de comunicación, mayor tasa de fecundidad y explotación de trabajo infantil, además que las mujeres tenían peor calidad de vida mental.

En general, este estudio demostró que las principales variables de asociación se refieren a los aspectos sociodemográficos que afectaron las habilidades de lectura y aritmética y el PCS y MCS de los usuarios ribereños de la APS ribereña.
